# Sex-specific site preferences in response to resource value in *Drosophila prolongata*

**DOI:** 10.1007/s00265-026-03731-7

**Published:** 2026-05-08

**Authors:** Swarnima Mukherjee, Alessio N. De Nardo, Stefan Lüpold

**Affiliations:** 1 Department of Evolutionary Biology and Environmental Studies, University of Zurich, Zurich, Switzerland; 2 Department of Integrative Biology, Michigan State University, East Lansing, MI USA

**Keywords:** resource-distribution, territoriality, territory, aggression, mating status, preferences

## Abstract

In species with resource-based mating systems, males and females often differ in their spatial distributions, reflecting divergent reproductive strategies and associated fitness benefits. Using automated tracking, this study investigated the influence of resource distribution and quality on sex-specific spatial positioning and behavioral patterns in the fruit fly *Drosophila prolongata*. Both sexes exhibited a positive correlation between occupancy and the size and quality of resource-dense areas (RDAs), but their behavioral responses varied by sex and reproductive status. Many males occupied and aggressively defended large, high-quality RDAs despite the potential costs, particularly when competitors were abundant. Conversely, female spatial behavior was dependent on their mating status: unmated females spent little time in RDAs, likely due to elevated risks of male harassment, yet frequently visited large RDAs specifically for mate acquisition. After mating, females significantly increased their presence in large RDAs. Our findings elucidate how resource distribution can shape reproductive strategies and resultant spatial patterns for males and females in resource-based mating systems.

**Supplementary Information:**

The online version contains supplementary material available at https://doi.org/10.1007/s00265-026-03731-7.

## Introduction

The spatial distribution of organisms is determined by a confluence of environmental variables, intrinsic individual traits, and social interactions. In many species, the spatial arrangement of individuals is strongly linked to the availability of critical resources such as food (Bro-Jørgensen et al. 2008; Tableau et al. 2016) or conspecifics (Bro-Jørgensen et al. 2008; Folmer and Piersma 2012). Particularly in species with resource-based mating systems, males and females may differently occupy space due to their disparate interests and reproductive strategies. Males may establish territories encompassing high-quality resources to maximize mating opportunities, whilst females may be attracted to these same locations for oviposition, foraging, or the presence of suitable mates. Conversely, certain individuals of either sex may also avoid such areas, e.g. to mitigate the costs of territoriality or intrasexual competition for males (Ord 2021) or to evade male harassment for females (Darden and Croft 2008). Understanding the spatial distribution of males and females relative to resource availability can provide critical insights into the mating system of a species, the distinct reproductive strategies adopted by each sex, and the potential for varying intensities of sexual selection resulting from fluctuations in local population densities or operational sex ratios (OSRs). The spatial configuration of resources alone can alter the traits under sexual selection by determining whether resources can be defended as a singular territory or multiple discrete units (Reichard et al. 2009).

The impact of resource distribution on individual spacing is not uniform. Heterogeneously distributed resources, including food, nesting sites, or receptive mates, are susceptible to monopolization by competitively dominant individuals, leading to the emergence of territoriality (Brown 1964; Emlen and Oring 1977; Grant 1993). However, active territorial defense incurs costs, potentially including physical injury, increased mortality risk, reduced foraging efficiency, elevated energetic expenditure, or heightened susceptibility to predation (reviewed in Ord 2021). Consequently, the viability of territorial defense depends on *economic defensibility* (Brown 1964). First, an increase in *competitor density* elevates the costs and diminishes the efficacy of defense, whereas exceedingly low competitor densities may render resource defense superfluous. Second, *resource distribution* plays a pivotal role: while aggregated resources are more amenable to defense compared to uniformly distributed resources, excessive concentration can attract an overwhelming number of competitors. Third, the nature of the resource and an individual’s condition, including its *resource holding potential* (RHP; Parker 1974), can determine defensibility. The confluence of these factors ultimately dictates the net fitness benefits associated with territoriality (Grant 1993). For example, in the convict cichlid (*Archocentrus nigrofasciatus*), males defending territories of intermediate size grow faster than those defending larger, more resource-abundant territories, likely due to the physiological stress associated with constant defense (Praw and Grant 1999). Similarly, *Drosophila melanogaster* males primarily defend territories when females are present and the territory is both sufficiently small for effective defense yet sufficiently attractive to females (Hoffmann and Cacoyianni 1990).

Analogous to males, the costs and benefits of spending time in resource-rich areas also influence female spatial distribution. The processes of mate searching and assessment are energetically costly (Parker 1983; Pomiankowski 1987; Reynolds and Gross 1990), but these costs may be mitigated if territoriality on high-quality resources serves as a reliable cue for male quality (Candolin and Voigt 2001; Deb and Balakrishnan 2014). Choosing a male with greater territorial success could also provide a shortcut to acquiring a high-quality mate with superior stamina through passive choice mechanisms (Kokko et al. 1999; Castellano 2009; Kovalov and Kokko 2022), as illustrated in the dragonfly *Diastatops obscura*, where females commonly accept the first territorial male encountered (Irusta and Araújo 2007). Beyond reducing mate choice costs, females may accrue indirect fitness benefits for her offspring by selecting high-condition mates (Candolin and Voigt 2001; Seddon et al. 2004; Vanpé et al. 2007; White and Rundle 2015). However, female attraction to territories is not solely driven by mating opportunities, as males frequently defend resources that are essential or directly beneficial, such as food, breeding/oviposition sites, or protection from predators (Verner and Willson 1966; Carranza et al. 1990; Calsbeek and Sinervo 2002; Suzuki et al. 2006; Dijkstra et al. 2008; Verdolin and Slobodchikoff 2010; Tregenza et al. 2022), to the extent that female competition for access to males and the resources they control can occur, as documented in certain mammals (Stockley and Bro-Jørgensen 2011). While females may be attracted to these resources, high male densities often result in harassment, potentially leading some females to select less favorable habitats (Bauer et al. 2005; Darden and Croft 2008; Bacon and Barbosa 2020). Competition from conspecific females can further discourage resource utilization, particularly if it escalates into aggressive interactions (Bath et al. 2017, 2021) or intensifies juvenile competition at oviposition sites, both interspecifically (Resetarits Jr and Wilbur 1989; Chess et al. 1990; Kidera and Takahashi 2020) and intra-specifically (Kanno and Harris 2002; Munga et al. 2006; Elsensohn et al. 2021). Given this complex interplay of factors, discerning whether female presence or absence at a resource patch is primarily driven by mating opportunities, nutritional needs, or the avoidance of harassment and competition can be challenging.

A similar ambiguity exists for males: it is often not clear whether males defend territories they perceive as intrinsically valuable and await female arrival, or whether they defend resources around which females are already aggregated. The predominant scenario likely varies across species. A study in the strawberry poison frog, *Dendrobates pumilio*, suggests that while males appear to defend tadpole-rearing sites to facilitate access to females, the data indicate that females are attracted to these sites, and male distribution subsequently follows female distribution (Pröhl and Berke 2001). Additionally, females tend to use tadpole-rearing sites not associated with their mate’s territory (Pröhl and Berke 2001). In contrast, in the treefrog *Kurixalus eiffingeri*, females showed no preference for intrinsic characteristics of oviposition sites but rather oviposited in sites selected by a territorial male (Poo et al. 2024).

Here, we explored the distribution and behavior in relation to resource size and quality in males and females of the South-Asian fruit fly *Drosophila prolongata*. This species is native to Southeast Asian forested highland regions, including southwestern China, northeastern India, Myanmar, and northern Vietnam (Singh and Gupta 1977). While no recorded observation of *D. prolongata* on their natural food source is known, wild flies have been successfully baited using banana and yeast traps (Achumi et al. 2016; Perdigón Ferreira and Lüpold 2022) and are attracted to various fruit-juice-based foods in the lab. Amino and Matsuo (2023) suggest that “males of this species occupy the top surface of small fruits on the ground” (p. 3), which supports the hypothesis that *D. prolongata* are likely generalist fruit breeders like most species in the *Drosophila melanogaster* clade (Ishikawa et al. 2022). In these fruit-breeding species, the size and distribution of food patches modulate their attractiveness and the intensity of local competition for males and females (Hoffmann and Cacoyianni 1990; Churchill et al. 2020, 2021).

*Drosophila prolongata* exhibits a pronounced male-biased sexual dimorphism in body and foreleg size (Setoguchi et al. 2014), unique among the Drosophilidae (Rohner et al. 2018). Males utilize their forelegs to engage in intense intrasexual combat over resources (Kudo et al. 2015; Amino and Matsuo 2020, 2023; De Nardo et al. 2025a). Successful occupancy of a territory increases male mating success under an even, but not a male-biased operational sex ratio (OSR; De Nardo et al. 2025a). However, under a male-biased OSR, smaller males appear to gain a competitive advantage (De Nardo et al. 2025b), potentially indicating a shift toward a scramble competition mating system (Moya-Laraño et al. 2007; Herberstein et al. 2017; Kelly 2020), when territorial defense becomes less effective. Depending on their individual condition, males also adopt an alternative mating tactic by intercepting other males’ mating attempts (Perdigón Ferreira and Lüpold 2022). Thus, while resource defense appears to be the primary strategy, the specific tactics employed can vary depending on the social context and individual condition.

We recorded the behavior of groups of male and female *D. prolongata* in arenas containing two resource-dense areas (RDAs) under an even OSR. Each arena comprised one large and one small RDA but varying in quality in a full-factorial design. Using the deep-learning based tracking software SLEAP (Pereira et al. 2022), we tracked the spatial coordinates of each individual across frames, acquiring detailed information on their distribution relative to resources of varying size and quality, as well as their sex. We also trained a random forest classifier to predict courtship, aggression, and mating behaviors based on features extracted from the tracked coordinates of individuals and their body parts. This paper focuses on the distribution of individuals and their attraction to resources as a function of resource size and quality, as well as the individuals’ sex, mating status, and surrounding competitors or mates. We also investigate the economic defensibility of resources and the role of aggression in securing access to territories. A separate paper, based on the same experimental dataset, focuses on sexual selection in relation to territoriality and condition-dependent reproductive strategies in either sex (De Nardo et al. 2026).

Here, we tested sex- and status-specific responses to resource size and quality against a baseline null hypothesis of random space use, in which RDA occupancy scales with patch area alone (i.e., proportional occupancy) and shows no additional attraction to high-quality food. Departures from this null would support active resource preference and/or socially mediated aggregation, as opposed to passive sampling of space (area-only effects) or density-driven scramble competition. Specifically, we predicted that both sexes would preferentially occupy large and high-quality RDAs beyond area effects, but that males would allocate more time to RDAs than females due to the fitness benefits of resource control. We further predicted that male occupancy would be mediated by aggressive interactions: males showing higher aggression should achieve higher RDA occupancy, and aggression should increase with competitor density and female presence. Because large and/or high-quality RDAs are expected to attract more individuals, we predicted reduced economic defensibility of these patches (higher aggression, fewer cases of exclusive dominance). For females, we predicted mating-status-dependent space use: unmated females should preferentially visit large/high-quality RDAs for mate acquisition, whereas mated females should reduce use of male-dense RDAs to avoid harassment (alternative explanations include attraction to nutrition/oviposition resources rather than mating opportunities, or weak harassment effects). Finally, we tested whether the ability to exploit both RDAs is associated with high mobility, consistent with an opportunistic tactic, versus the alternative that dual-patch occupancy is rare or unrelated to movement.

## Materials and methods

### Study organisms

The null specimens used in this study originated from a genetically diverse outbred population collected from the Sa Pa region of Vietnam in 2018. Since then, this population has been maintained under laboratory conditions at 20 ± 1 °C and 60% relative humidity, on a 14:10 light: dark cycle. To generate the experimental cohort, eggs were transferred to food vials (60mL volume, containing 12mL of food medium) at standardized density of 20 eggs per vial. To introduce variation in adult body size, food was provided in three distinct qualities (high quality: 55 g corn, 100 g yeast, 75 g saccharose, 8 g agar, 10 g flour, 15mL Nipagin per liter; medium quality: diluted by ½ in water and agar; low quality: diluted by one 1/4). While body size was manipulated, it is not a variable considered within the scope of this paper but was used to investigate condition-dependent reproductive strategies in a related study (De Nardo et al. 2026) based on the same experimental trials. Upon eclosion, males and females were separated by aspiration within 24 h to ensure virginity and were subsequently maintained in single-sex groups of 5 males or 15 females, respectively.

### Experimental setup

At least two days before the experimental trials, each fly was marked on its thorax with a unique color dot using acrylic paint (Kreul El Greco Acrylic colors) under brief ice anesthesia. Each color was exclusively used once per sex within each experimental arena. After marking, each individual was housed in isolation until the start of the experimental trials. Each experimental group of flies was introduced into a Petri dish (ø 10 cm) with both a small and a large resource-dense area (RDA) to create variation in resource distribution (Fig. S1). The large RDA was a rectangular plastic container (2.2 [L] ⋅ 2.2 [W] ⋅ 0.8 [H] cm; Semadeni, Switzerland), covering approximately 7.5% of the total area, while the small RDA consisted of the cap of a 5mL Eppendorf tube (ø 0.9 cm; approx. 1.3% of the total area) elevated to the same height as the large RDA using non-toxic putty. Both RDAs were filled to their capacity with fly medium (same as ‘high quality’ above). The remaining area of the Petri dish was filled with orange juice agar (600mL water, 200mL orange juice, 18 g agar, 20 g saccharose, 20mL Nipagin for approx. 800mL) up to the rim of the RDAs. To introduce variation in resource quality independent of RDA size, the fly medium in each RDA was either covered with yeast paste (approximately 1:1 ratio of dry yeast and orange juice) to represent a high-quality resource or sprinkled with dry yeast for a nutritionally lower-quality RDA. While each arena contained a large and a small RDA, quality treatments were either both high, both low, or one of each (either small or large; i.e., four different RDA treatments). To each Petri dish we introduced eight males and eight females and then recorded them using a Raspberry Pi equipped with a camera module (V2) mounted directly above the Petri dish, all inside a climate chamber at 20 ± 1 °C and 60% relative humidity. Total sample for males and females were 320 individuals each. However, only 319 males were included in the analysis, as a male escaped during the recording and had incomplete data. Flies from different developmental treatments were haphazardly assigned to Petri dishes. Images were captured every two seconds for a duration exceeding 30 min. A total of 10 replicate arenas (with distinct groups of flies) were recorded for each of the four RDA quality treatments. Half of the recordings for each treatment were conducted in the morning (between 10:00 and 12:00 h), and the other half in the afternoon (between 14:00 and 16:00 h). Similarly, within each quality treatment, half of the arenas were positioned under the camera with the large RDA oriented toward the right, and the other half reversed to account for potential effect of side biases due to external factors. The recordings were performed across eight camera setups over five days, with two arenas of each RDA quality treatment recorded simultaneously.

### Processing of recordings

The image sequences captured during the first 30 min of recording were converted to video format (mp4) and subsequently analyzed using the deep-learning software SLEAP version 1.3.0 (Social LEAP Estimates Animal Poses; Pereira et al. 2022). The SLEAP model was trained on manually labeled training sets (Fig. S2) over four iterative training rounds, with the addition of more labeled frames to the training set in each round (Fig. S3; Table S1), culminating in a total of 1,600 training frames (= 25,600 individual instances) and a constant validation set of 160 frames (= 2,560 instances). We used the multi-animal top-down approach, using a combination of a centroid model for initial instance detection and a centered instance model for subsequent body part localization (Pereira et al. 2022; details on training settings in Table S2). The model was trained to identify the head, thorax, and both wings of both males and females. The resulting trained model demonstrated high accuracy in identifying individual flies and their body parts (see Table S3; Fig. S4) and so was therefore used to track flies across ten videos for each RDA quality combination, resulting in 40 tracked videos (detailed tracking settings are provided in Table S4). Errors in cross-frame tracking, such as identity switches between individuals, were manually corrected through systematic frame-by-frame inspection.

### Processing of extracted data & automated categorization of behavior

The tracking data generated by SLEAP was exported in hdf5 format. Regions of interest (ROIs) were manually defined in Fiji (Schindelin et al. 2012), encompassing the RDAs and their immediate surroundings, and were saved for later analysis. To standardize the probability of an individual randomly being present in RDAs of different sizes, we computationally added a buffer zone around the small RDA to equalize its total area with that of the large RDA (Fig. S1). This was achieved by measuring the area covered by the large RDA in each arena using Fiji and then creating a circular ROI centered on the small RDA with an equivalent area. This procedure was implemented in step 3) of the custom Python pipeline outlined below, during the assignment of pixels to ROIs. Consequently, observed differences in RDA occupancy can be attributed to the variation in resource size and quality rather than inherent biases associated with ROI sizes. This was also further controlled for using the Jacobs’ Index (Jacobs 1974; Cock 1978), which is described in more detail in the statistical methods.

A custom-built Python pipeline (Table S5-6) was developed and implemented for data processing and analysis, with the goal to (1) extract the spatial coordinates of individuals from HDF5 files generated by SLEAP; (2) interpolate missing data on thorax coordinates (based on the positions of other visible body parts, or if necessary, through linear interpolation; representing 1–4% of all thorax coordinates per experimental unit, primarily occurring when flies were positioned upside down on the arena lid with the thorax obscured), as the thorax position was a key variable in downstream calculations; (3) delineate the ROIs (i.e., RDAs) based on pixel coordinates; (4) determine whether the body parts of individual flies were located within defined ROIs by comparing their pixel coordinates; (5) calculate a suite of behavioral features derived from the tracked body part coordinates, including wing angles, velocities (distance moved between consecutive frames), distance to the nearest male/female conspecific, male gaze direction, whether an individual’s gaze was directed toward any or the nearest male/female, and the temporal changes in these features. A comprehensive list of these and additional features (e.g., change in gaze direction or “turning angle”) are provided in Table S8.

Using the Python library *scikit-learn* (Pedregosa et al. 2011; Varoquaux et al. 2015), a random forest classifier was trained on the different behavioral categories such as mating, courtship, and aggression (note, however, that courtship was not used in this paper but in (De Nardo et al. 2026), which is based on the same experiment). A random forest classifier is a powerful machine learning algorithm that constructs multiple decision trees on different subsets of the training data to identify the features most predictive of the categorical outcomes (Hao and Ho 2019). To this end, a training data set was created for each behavioral category, based on visual coding of the behavior of 64 males across 200–400 frames in each of eight experimental units (representing two replicates for each RDA quality combination), resulting in 19,244 behavioral observations and 138 extracted features. To ensure a balanced training dataset for each behavior, each instance of the target behavior was matched with an equal number of randomly selected frames where that behavior was not observed. This approach resulted in balanced datasets of 6,832 observations for mating, 4,206 observations for courtship, and 4,208 observations for aggression, respectively. For independent validation during the training process, a stable subset comprising 20% of the dataset was reserved, while the remaining 80% were used for model training.

To optimize the hyperparameters of the random forest classifier, an extensive grid search with k-fold cross-validation (threefold) was conducted for each of the behavioral categories. This iterative process systematically narrowed down the parameter space to identify the optimal model configuration for each behavior (Table S7). Additionally, recursive feature elimination (RFE) was used to reduce the dimensionality of the feature space from the initial 138 to the optimal number for each behavior (Table S8-9), thereby excluding irrelevant features and mitigating the risk of overfitting. Each permutation of the grid search and RFE (with progressively refined parameter ranges and feature subset sizes based on previous iterations) was conducted 100 times with a different random seed in each iteration, while consistently using the same validation subset to ensure comparability across runs. After four such permutations, features that were consistently excluded in the majority of the iterations were removed from the dataset until the optimal number of features was reached for each behavioral model. The final random forest models were trained using the optimal parameters identified during the grid search and the reduced set of relevant features. The performance of the trained random forest classifiers for each of the behaviors was high, achieving accuracies of 98.02% for mating, 93.11% for courtship, and 94.06% for aggression (Table S10).

Post prediction, mating frame sequences were checked for potential prediction inaccuracies. Due to occasional imperfect prediction, gaps within actual mating sequences were filled based on the following rules: single-frame gaps surrounded by mating frames were immediately filled. Subsequently, gaps of two to ten consecutive frames were filled if > 50% of the ten frames preceding and 10 succeeding frames were classified as ‘mating’. Given that copulations in *D. prolongata* typically last for at least three minutes (with only 1.4% shorter durations observed in De Nardo et al. 2024), only corrected sequences with a minimum of 80 frames were accepted as valid mating events. This correction was validated on two experimental units of each treatment (= eight units), in which none of the 47 detected matings were incorrectly identified (precision = 1.0), and only one of 48 actual matings was missed (recall = 0.979). Furthermore, to ensure high accuracy, all detected mating events lasting for < 100 frames were visually inspected. During this manual verification, four out of a total of 233 detected matings (1.7%) were misclassified.

After correcting all mating instances, any courtship or aggression bouts that were falsely predicted during confirmed mating sequences were removed, as mating males cannot simultaneously display courtship or aggression while mating. Finally, the accuracy of the predicted courtship and aggression behaviors were re-evaluated on 20 randomly selected frames from one experimental unit of each treatment (*n* = 1,280 observations) that were not included in the training dataset. For each of these frames, the behavior of each male was visually assessed and then compared to the prediction generated by the random forest classifier. This independent validation revealed an accuracy of 98.6% for courtship and 98.5% for aggression.

### Variable definitions

The variables and behaviors are defined, and their quantification explained in Table 1.

**Table 1:** Definitions and quantification of behaviors and variables used in the analysis

Variable/Behavior	Definition	Quantification
Occupancy	*Behavior*: Whether an individual is located in an RDA *Variable*: Occupancy as used in the analyses is the proportion of frames an individual occupies an RDA in a given period (e.g., overall or per minute). In this study, we excluded the time *in copula* from measures of occupancy, except where stated differently.	*Behavior*: Thorax coordinate inside of predefined region of interest in that frame *Variable*: Proportion of occupancy frames out of total observed frames. In this study, the frames during which an individual was *in copula* (i.e., detected as mating frame) were excluded from the occupancy calculation.
Exclusive occupancy	*Behavior*: Male occupies RDA without any other males in the RDA. *Variable*: Proportion of frames with only one male in RDA out of total frames with non-zero males in RDA.	*Behavior*: Only one male fulfills criteria for occupancy of an RDA in a given frame *Variable*: Proportion of frames out of those with non-zero males fulfilling the criteria of occupancy with only one male fulfilling the criteria of occupancy.
Access	*Behavior*: Entering an RDA *Variable*: Number of accesses	*Behavior*: Crossing the predefined outline of a region of interest from outside to inside (occupancy 1 in current frame, but 0 in previous frame) *Variable*: Count of accesses
Mobility	*Per frame*: Distance moved since previous frame *Variable*: Mean distance moved between frames within given period (e.g., overall or per minute)	*Per frame*: Vector length of the frame-to-frame displacement in the x- and y-coordinates of the thorax (or head if thorax is not available) between frames in pixels *Variable*: Mean vector length within given period
Aggression	*Behavior*: Established agonistic behaviors between individual *Drosophila* males, including chasing, fencing, lunging, tussling and boxing, not including threats (e.g., Chen et al. 2002; Jonsson et al. 2011; Zwarts et al. 2012; Belenioti and Chaniotakis 2020). Ethogram in Table S11. All of these were considered aggressive behavior. *Variable*: Proportion of frames in which aggressive behavior was observed out of total observed frames in a given period (i.e., aggression rate)	*Behavior*: Manual labeling of training data frames following the ethogram (Table S11) for behavioral categorization by random forest classifier. Trained model predicts instances of aggressive behavior on frame by frame level for each male. We followed the ethogram to decide whether a behavior is aggressive, but did not differentiate the different types of aggressive behaviors for training. *Variable*: Proportion of frames for which aggressive behavior was predicted by random forest classifier out of total observed frames in a given period (i.e., aggression rate)

### Statistical analysis

The data were analyzed and visualized in R version 4.3.1 (R Core Team 2023). Heat maps of spatial distributions were created by combining the thorax coordinates of all individuals across all frames in one file per sex. The coordinates were then aligned relative to the midpoints of the two RDAs to obtain comparable distributions. We employed the *stat_density_2d* function from *ggplot2* (Wickham 2016) to plot the density contours of the thorax coordinates, and overlaid the reference shapes (outlines of the arena and RDAs).

To account for differences in RDA area and the unequal probability of occupying RDAs by chance alone, we added a buffer zone around the small RDA such that its total area matched that of the large RDA. This enabled comparisons between RDAs of equal area. Unless stated otherwise, references to the small RDA include this buffer zone. Additionally, to quantify individual preference for RDAs while accounting for unequal area sizes, we calculated Jacobs’ Index of preference (Jacobs 1974; Cock 1978). For each individual and RDA, the observed proportion of time spent within the RDA was compared to the expected proportion based on its relative area, yielding an index ranging from − 1 (complete avoidance) to + 1 (complete preference), with 0 indicating use proportional to availability. Jacobs’ Index was calculated separately for each RDA size and individual using the proportion of frames in which the individual’s thorax was located within the respective region of interest. The index was calculated as *D* = (*u* − *a*)/(*u* + *a* − 2*ua*), where *u* is the observed proportion of use and *a* is the expected proportion based on area availability.

In addition to models on averaged predictors and outcome variables across the entire 30-minute observation period, we summarized the data per individual in 1-minute time bins (i.e., 30 bins in total) to identify more detailed causal relationships between predictor variables and occupancy. Linear mixed effects models (LMM) were conducted using the package *lme4* (Bates et al. 2015), while binomial repeated-measures data (e.g., proportion of occupancy frames out of 30 total frames per 1-minute bin) were analyzed using betabinomial models as implemented in the *glmmTMB* package (Brooks et al. 2017), to mitigate overdispersion. All models included the unit (experimental arena) as a random effect, whilst repeated-measures models (i.e., those using long format with multiple observations per individual) additionally included individual ID (nested within unit) and the time bin as additional random effects. Model performance was validated using the *DHARMa* package (Hartig 2016).

We included RDA size and quality, as well as either hypothesized predictors or control variables in each model (depending on the sex). Interactions were added as necessary based on our hypotheses, but due to very large sample sizes (*n* > 15,000 observations) in the repeated-measures analyses, we kept interactions in the models based on the Akaike information criterion (ΔAIC ≥ 2) rather than based on *P*-values, to avoid including significant yet insubstantial interactions. To quantify and visualize adjusted effects from the fitted models, we used estimated marginal means as implemented in the *emmeans* package (Lenth 2023).

Model-based predictions were computed on the link scale and subsequently back-transformed to the response scale to obtain predicted probabilities. Continuous covariates were held at their median values and categorical predictors were averaged over according to their observed proportions. Adjusted effect sizes were expressed as differences in predicted probabilities (Δ response). For continuous predictors, biologically meaningful contrasts were defined as differences between the 25th and 75th percentiles of the observed data, representing a large but not extreme range. In addition, odds ratios for binomial models were obtained by exponentiating fixed-effect estimates, and standardized effect sizes (partial *r*) were calculated from model statistics using the *effectsize* package (Ben-Shachar et al. 2020). 95% confidence intervals were derived from the model-based estimates on the response scale, and *P*-values were adjusted using the Holm correction where multiple comparisons were performed on the same variables. Models outcomes were visualized using *ggplot2* (Wickham 2016) based on predictions made with *ggeffects* (Lüdecke 2018).

## Results

### Distribution relative to RDAs

To compare the overall occupancy of resource-dense areas (RDAs) between males and females, quantified as the proportion of frames spent in an RDA (Table 1), we summed for each individual the time spent in the large and small RDAs (including or excluding the buffer zone around the small RDA). A betabinomial GLMM (*n* = 639, i.e., 319 males and 320 females) revealed that, regardless of whether the buffer zone around the small RDA was included, males spent more time in RDAs than females (without buffer: Δ occupancy_*females−males*_ = 0.051 [0.018, 0.084] vs. with buffer: Δ occupancy_*females−males*_ = 0.053 [0.016, 0.088]; both *z* ≥ 2.82, *P* ≤ 0.003; Table S12, Fig. S6). Notably, when the frames during copulation were omitted (only for individuals *in copula*), this effect became more pronounced (without buffer: Δ occupancy_*females−males*_ = 0.068 [0.035, 0.101] vs. with buffer: Δ occupancy_*females−males*_ = 0.069 [0.032, 0.105]; both *z* ≥ 3.66, *P* < 0.001; Table S12; Fig. S6). Given that 53.2% of observed copulations started within an RDA (41.6% in the large RDA and 11.7% in the small RDA including its buffer), inclusion of mating events may overestimate the time spent on RDAs, particularly for females. Moreover, even at the end of copulations, a proportion of pairs had already moved outside of RDAs, with only 40% of copulations ending within an RDA (31.4% in the large RDA, and 8.6% in the small RDA including its buffer). Consequently, as the primary focus of this study was on the distribution and movement patterns relative to RDAs, individuals *in copula* were considered in a “time-out” state (i.e., not actively competing over resources). Thus, analyses on territorial occupancy were restricted to non-mating phases for both sexes.

**Fig. 1:**
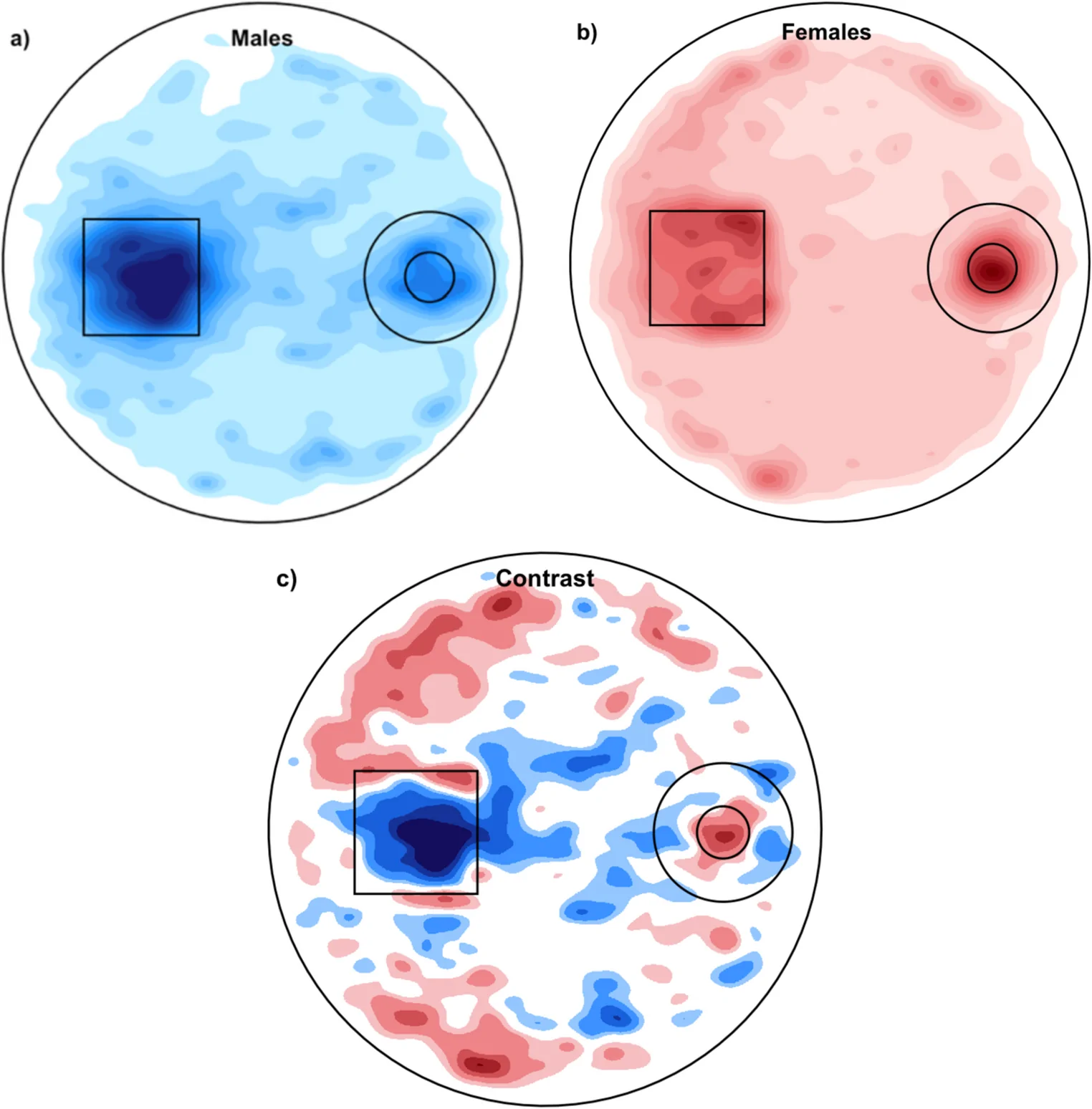
Spatial distribution of non-copulating (**a**) males and (**b**) females across all treatments, with the square representing the large RDA, and the double-circle depicting the small RDA and its buffer zone. Darker colors indicate higher densities of observations among the eight individuals per sex across the 40 experimental arenas. Panel (**c**) illustrates the male-female contrast, with blue signifying more male than female observations, white indicating similar observation levels, and red reflecting female-biased observation

Based on this definition of occupancy, we tested predictions that both sexes show a preference for large and high-quality RDAs, and that males allocate more time to RDAs than females. The full model was reduced from a four-way interaction to a model with a three-way interaction between RDA size, quality, and quality of the other RDA, with sex not involved in any interaction. Males spent more time on RDAs, regardless of other factors (Δ occupancy_*females-males*_ = 0.026 [0.013, 0.039], *z* = 4.07, *P* < 0.001; *n =* 1,917 [639 flies across 3 regions of interest], Table S13). For both males and females, a significant three-way interaction between RDA size, quality, and quality of the other RDA (*X*^2^_2_ = 18.74, *P* < 0.001) indicates that how much time is spent in an RDA depends on several factors. Across all quality conditions, large RDAs were consistently visited for longer periods than the small RDA (Δ occupancy_*small–large*_ > 0.101, *z* > 5.01, *P*_*adj*_ < 0.001) or its equal-sized buffer zone (Δ occupancy_*buffer-large*_ > 0.059, *z* > 2.64, *P*_*adj*_ ≤ 0.009) (Fig. 1). On the other hand, high-quality RDAs were only visited more than low-quality RDAs under certain conditions. Flies spent more time on large high-quality RDAs than large low-quality RDAs only if the small RDA in the same unit was of high quality (Δ occupancy_*low-high*_ = 0.078 [0.018, 0.14], *z* = 2.54, *P*_*adj*_ = 0.01), but not if the small RDA was of low quality (Δ occupancy_*low-high*_ = 0.043 [−0.022, 0.109], *z* = 1.29, *P*_*adj*_ = 0.20). Similarly, flies spent more time in a high-quality small RDA compared to one of low quality only if the large RDA was of low-quality (Δ occupancy_*low-high*_ = 0.033 [0.008, 0.059], *z* = 2.56, *P*_*adj*_ = 0.01) while quality did not matter for occupancy of the small RDA when the large RDA was of high quality (Δ occupancy_*low-high*_ = −0.012 [−0.033, 0.008], *z* = −1.18, *P*_*adj*_ = 0.24).

**Fig. 2:**
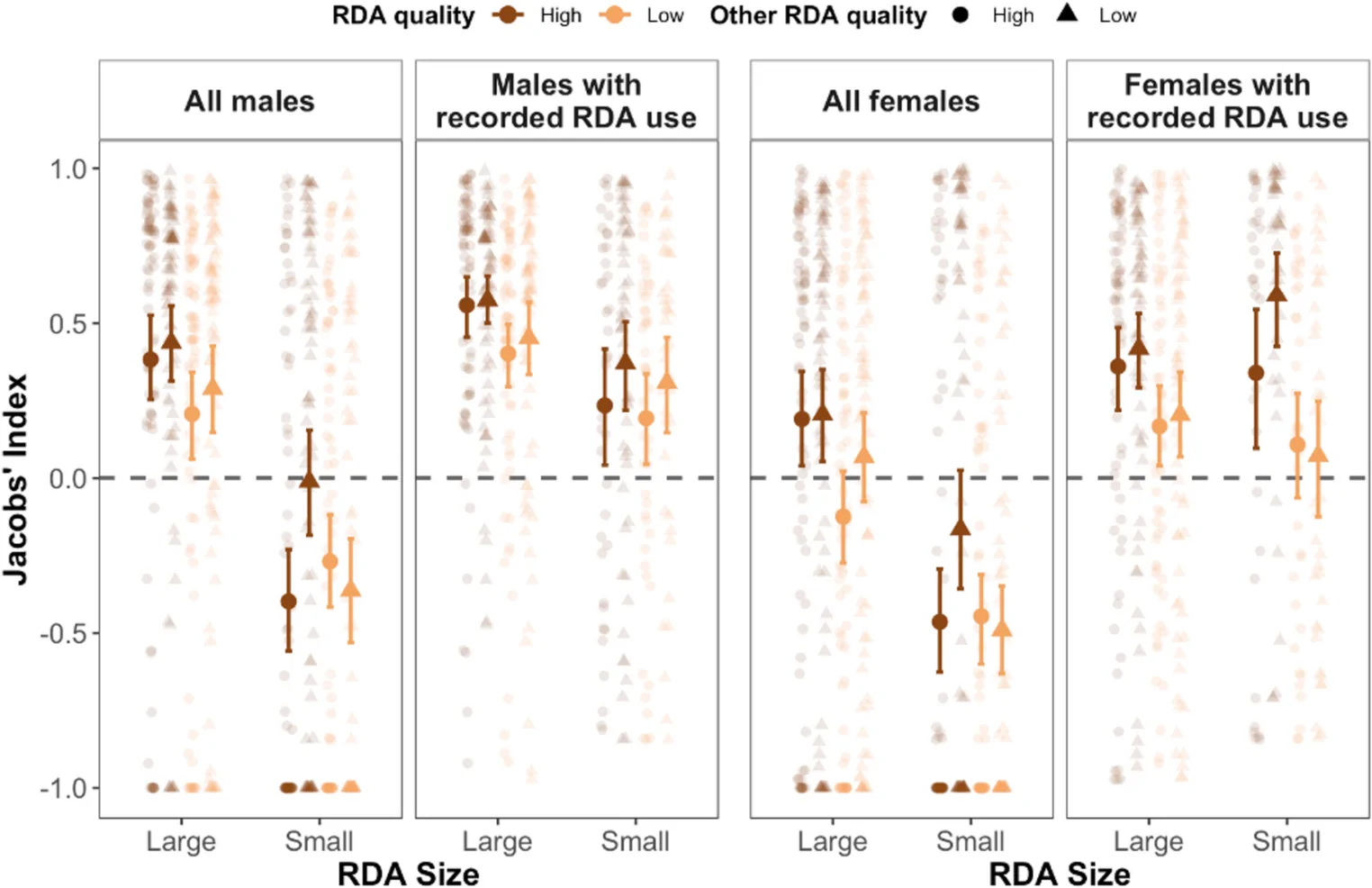
Jacob’s Index (mean ± bootstrapped 95% confidence intervals) showing preference for (> 0) and avoidance of (< 0) RDAs depending on RDA size, RDA quality, and quality of the other RDA. For each sex, the left plot shows the data across all individuals, while the right plot omits those who were never recorded in that RDA

For all subsequent analyses (except for the Jacobs’ Index analysis), the buffer zone was consistently included in the definition of the small RDA for direct comparability with the large RDA (although this will not be explicitly stated hereafter). We found a similar pattern for the Jacobs’ Index: males showed consistently positive values for large RDAs, indicating a preference for these areas, regardless of their quality and the quality of the other RDA (all Jacobs’ Indices ≥ 0.20, *t* ≥ 2.5, *P* ≤ 0.005). This pattern was mirrored by a negative bias against small RDAs across all males, as those values were negative across all combinations of RDA quality and alternative RDA quality in all but one case (Jacobs’ Indices ≤ − 0.26, *t* ≤ − 3.38, *P* ≤ 0.001), indicating avoidance of small RDAs relative to availability. The exception was the neutral outcome for high-quality small RDAs when the large RDA was of low quality (Jacobs’ Index = −0.01, *t* = −0.12, *P* = 0.90). After excluding those males that were never recorded on a particular RDA from the dataset, we found that preference for the large RDA increased within each quality category (confidence intervals did not contain means of the equivalent group; Fig. 2; Table S14). However, the difference made by the removal of the non-visitor males from the data had a much larger impact on the preference of the small RDA, for which all groups moved from negative or neutral Jacobs’ Indices to positive indices.

In females, Jacobs’ Indices showed a context-dependent pattern rather than a consistent size preference: Values for the large RDA were significantly positive only when the large RDA was of high quality, irrespective of the quality of the alternative RDA (Jacobs’ Indices ≥ 0.19, *t* ≥ 2.41, *P* ≤ 0.018). In contrast, Jacobs’ Indices for large low-quality RDAs did not differ significantly from zero when the alternative RDA was of high or low quality (|*t|* ≤ 1.61, *P* ≥ 0.11), indicating proportional use relative to availability. Consistent with this pattern, Jacobs’ Indices for small RDAs in females were consistently negative, particularly for low-quality small RDAs (Jacobs’ Indices ≤ − 0.45, *t* ≤ − 5.88, *P* < 0.001), indicating avoidance. Although mean female Jacobs’ Indices were negative for small RDA of high-quality RDAs under both alternative RDA quality conditions, it only differed from zero significantly when the alternative RDA was of high-quality (Jacobs’ Index = −0.45, *t* = −5.33, *P* < 0.001; with low alternative RDA: Jacobs’ Index = −0.17, *t* = −1.68, *P* = 0.098). However, similar to males, when non-visitor females were excluded, preference for large RDAs increased and became positive for low-quality RDAs as well (Jacobs’ Indices ≥ 0.17, *t* ≥ 2.39, *P* ≤ 0.02). Similarly, preference for small high-quality RDAs also became positive (Jacobs’ Indices ≥ 0.34, *t* ≥ 2.93, *P* ≤ 0.006), with that of small low-quality RDAs becoming neutral (*t* ≤ 1.25, *P* ≥ 0.22). Overall, these results indicate that female space use relative to RDA size was modulated by resource quality rather than exhibiting a uniform preference for large RDAs as shown for males. These differences in Jacobs’ Indices between all individuals of a sex and the subset that was recorded in an RDA at least once may indicate that the negative values observed across the full datasets does not imply general avoidance of the small RDA, but a likely group of individuals that never visits the small RDA due to a stronger attraction and access to the large RDA.

### Defensibility of RDAs

**Fig. 3:**
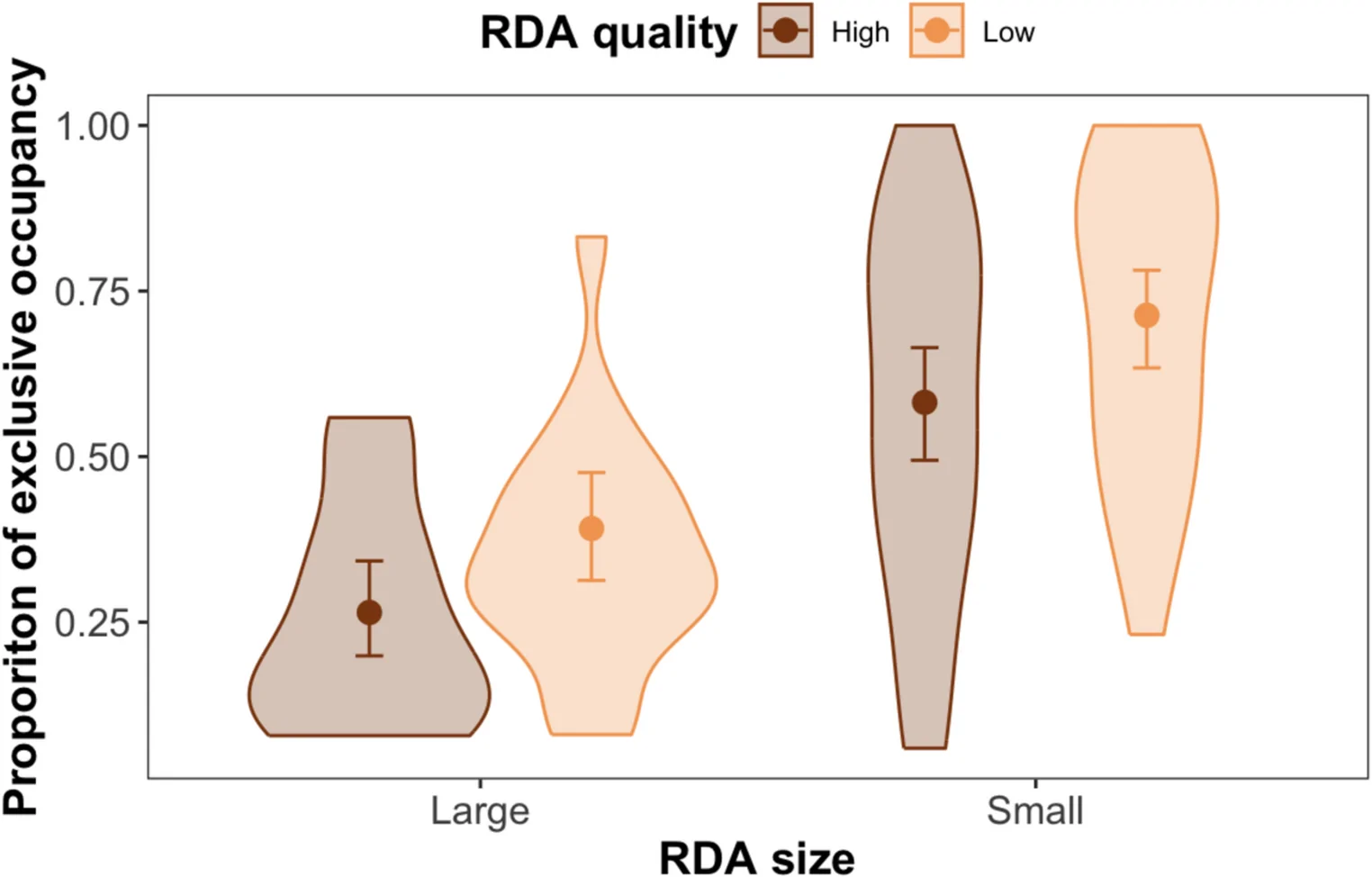
Proportion of exclusive occupancy (i.e., proportion of time in an RDA without a competitor; mean ± 95% confidence intervals) depending on RDA size and quality

Here, we tested the prediction that large and high-quality RDAs are more difficult to defend and show lower levels of exclusive occupancy. In an LMM (*n* = 80 [one observation per RDA per arena]), the average number of males present in an RDA (averaged across all frames) was significantly higher for large (Δ number of males_*small−large*_ = 1.09 [0.79, 1.39], *t* = 7.45, *P* < 0.001) and high-quality RDAs (Δ number of males_*low−high*_ = 0.44 [0.14, 0.74], *t* = 2.99, *P* = 0.004; Fig. S8; Table S15). Correspondingly, the exclusive occupancy (i.e., only one male present on an RDA) was greater for small (Δ exclusive occupancy_*small−large*_ = −0.33 [−0.42, −0.23]; *t* = 6.36, *P* < 0.001) and low-quality RDAs (Δ exclusive occupancy_*low−high*_ = −0.14 [−0.24, −0.04]; *t* = 2.77, *P* = 0.006; Fig. 3; Table S16), and the frequency with which individual males entered an RDA (i.e., total accesses per male) was higher for large (Δ accesses per male_*small−large*_ = 8.84 [6.91, 10.77], *z* = 11.71, *P* < 0.001; Table S17), but not high-quality RDAs (Δ accesses per male_*low−high*_ = 1.03 [−0.56, 2.61], *z* = 1.28, *P* = 0.20; Fig. S9).

### Factors determining occupancy

To elucidate the factors influencing RDA occupancy (defined as the proportion of time spent in RDAs), males and females were analyzed separately. The continuous predictor variables were standardized within each experimental unit and sex to enhance comparability across individuals and treatments (applied here and in the following analyses).

### Males

A betabinomial GLMM (*n* = 319) using variables averaged over the entire 30 min was used to test prediction (iii), that male aggression increases RDA occupancy: occupancy of the large RDA (occupancy-L) was positively correlated with male aggression (Δ occupancy_*25th−75th percentile*_ = 0.140 [0.099, 0.180]; *z* = 6.94, *n* = 319, *P* < 0.001; Table S18), while lower occupancy-L was observed in males exhibiting high (75th percentile) vs. low (25th percentile) occupancy of the small RDA (Δ occupancy_*25th−75th percentile*_ = −0.115 [−0.143, −0.088]; *z* = −8.55, *P* < 0.001) and in those with high (75th percentile) vs. low (25th percentile) mobility (Δ occupancy_*25th−75th percentile*_ = 0.048 [0.009, 0.087] *z* = 2.46, *P* = 0.014). Similarly, occupancy-S increased with male aggression levels (Δ occupancy_*25th−75th percentile*_ = 0.031 [0.009, 0.053]; *z* = 2.95, *P* = 0.003; Table S19) but was inversely related with occupancy-L (Δ occupancy_*25th−75th* percentile_ = −0.071 [−0.097, −0.046], *z* = −7.30, *P* < 0.001) and positively related to male mobility (Δ occupancy_*25th−75th percentile*_ = 0.019 [−0.001, 0.039]; *z* = 1.98, *P* = 0.048). Although aggression predicted occupancy of RDAs of both sizes, its effect on Δ occupancy was considerably larger for occupancy-L compared to occupancy-S (0.140 [0.099, 0.180] vs. 0.031 [0.009, 0.053]).

**Fig. 4:**
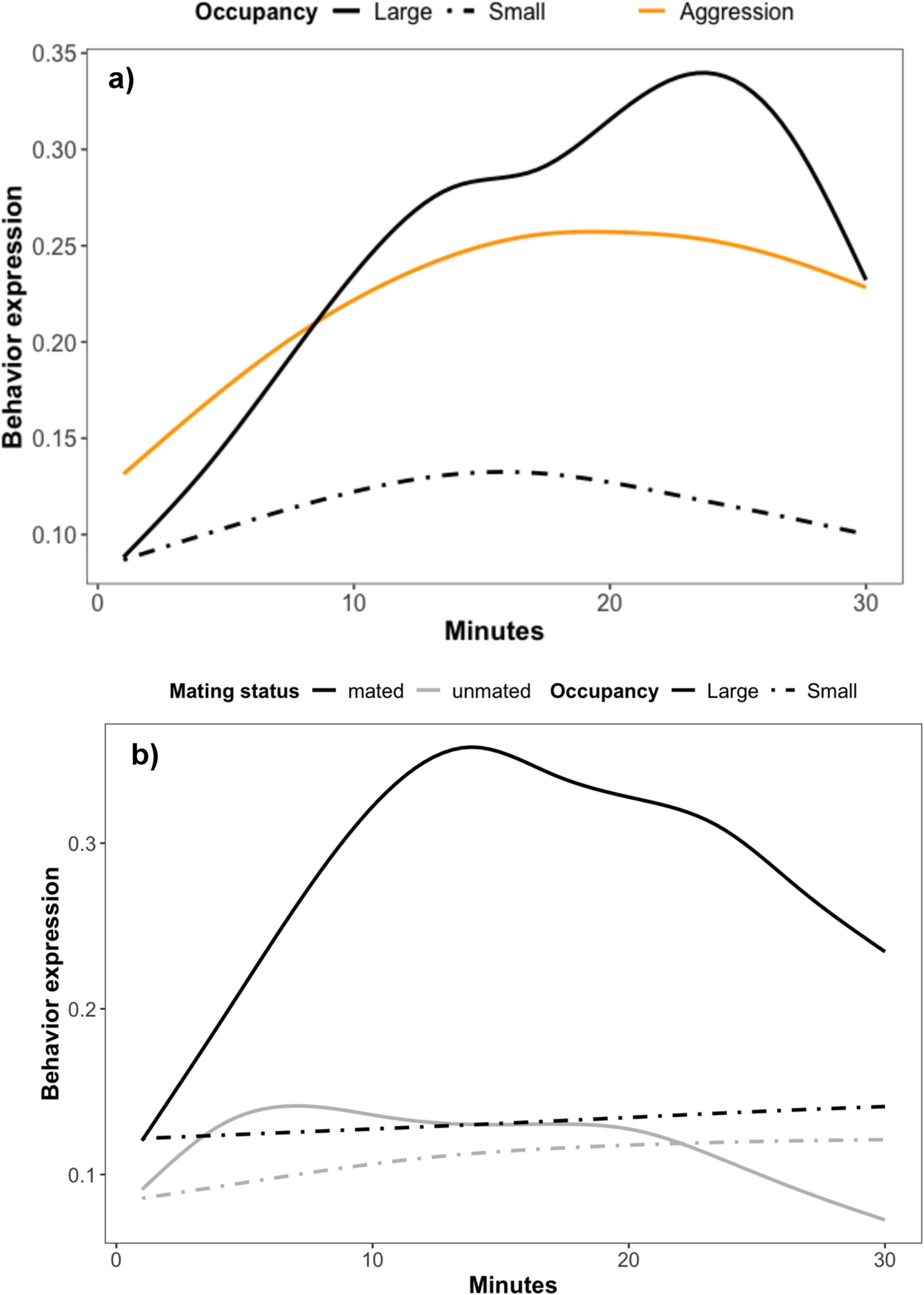
Change in **(a)** male occupancy of RDAs (in black; proportion of frames in RDA; small RDA dashed; large RDA solid) and aggression rates (in orange; proportion of frames showing aggressive behavior) over time **(b)** and female occupancy of RDAs (proportion of frames in RDA; small RDA dashed; large RDA solid) depending on their mating status (mated females in black; unmated females in grey). For both, the proportions were measured across one-minute intervals to visualize changes over time

The concurrent increase in aggression and occupancy-L over time suggests a potential causal relationship, which was not evident for the small RDA (Fig. 4a). To further test this and other time-dependent effects, we used a betabinomial repeated-measures GLMM with *n*_*tot*_ = 16,303 observations (30 one-minute intervals, 319 individual males, 2 RDAs [across 40 arenas], excluding 2,632 observations due to ongoing matings, and 205 observations excluded by the model due to missing data), where time and individual identity were included as random effects. We first tested the prediction that high mobility facilitates occupancy of multiple RDAs within a short period. We investigated the interaction between individual mobility and occupancy of the alternative RDA. As predicted, increased time spent on one RDA decreased occupancy of the other, with high-mobility males better able to maintain occupancy of both RDAs within the same one-minute period (*X*^2^_1_ = 99.33, *P* < 0.001). However, closer inspection of this interaction revealed that while it was statistically significant, the effect only occurred at a biologically irrelevant level of mobility. Occupancy of one RDA reduced occupancy of the other RDA equally at the 1 st percentile (Δ occupancy from 25th to 75th percentile of other RDA occupancy = − 0.145 [− 0.174, − 0.115]) and the 99th percentile of mobility (Δ occupancy from 25th to 75th percentile of other RDA occupancy = − 0.152 [− 0.186, − 0.118]); Fig. S10). Therefore, the interaction was removed from the model.

**Fig. 5:**
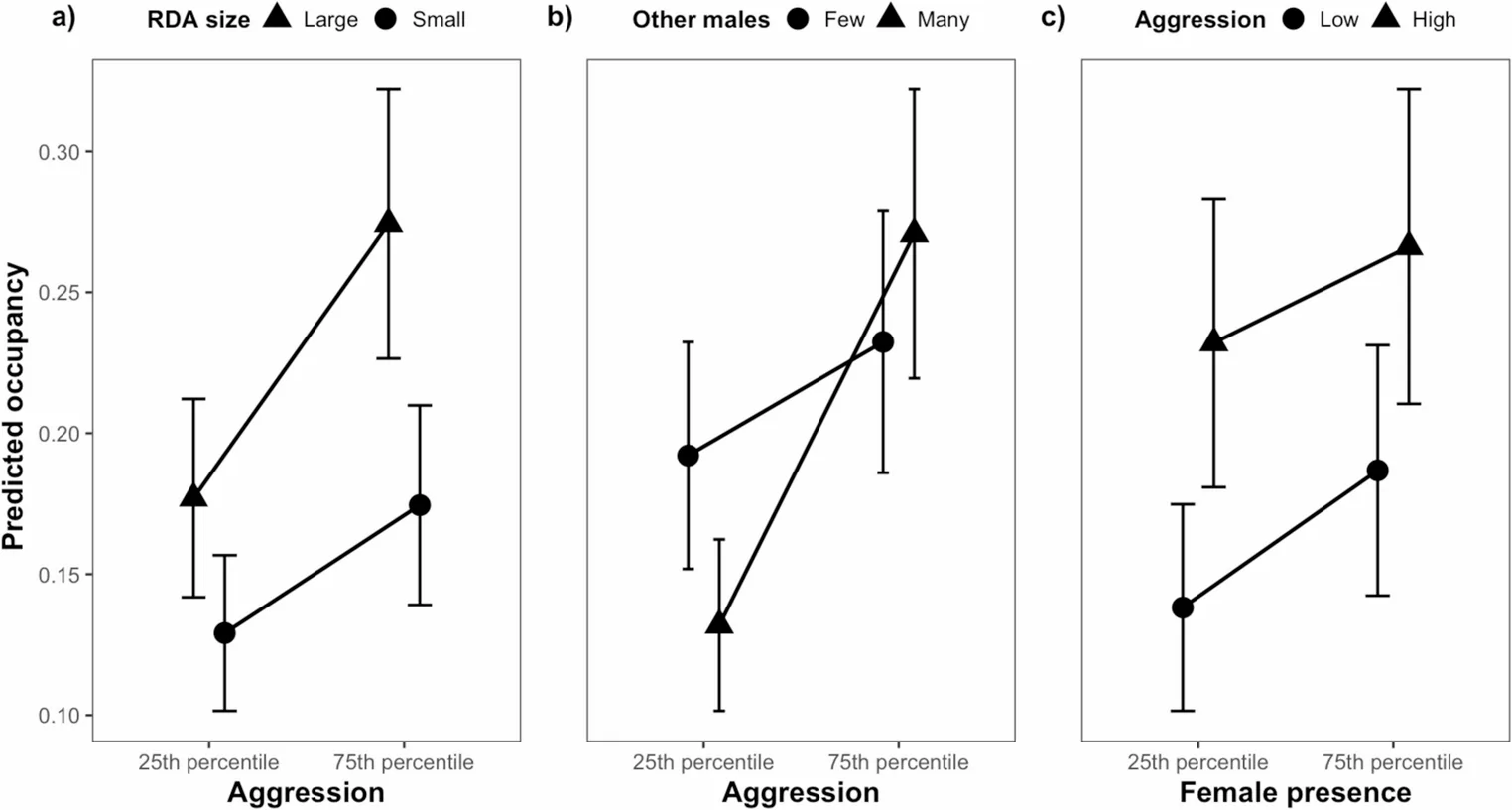
Male occupancy (i.e., proportion of frames spent in an RDA) were associated with: (**a**) higher aggression, particularly on large RDAs; (**b**) stronger positive effects of aggression at higher competitor densities (few: 25th percentile of competitor density; many: 75th percentile of competitor density); (**c**) increased female presence, especially among less aggressive males (low: 25th percentile of aggression; high: 75th percentile of aggression)

In the reduced model, we further tested the prediction that males should be more aggressive to gain occupancy, and increasingly so at higher competitor and female density. This analysis revealed that aggression had a significantly greater positive effect on occupancy-L than on occupancy-S (Δ occupancy_25th−75th percentile_ = 0.097 [0.080, 0.114] vs. 0.045 [0.032, 0.059], *X*^2^_1_ = 16.69, *P* < 0.001, Fig. 5a; Table S20). The effect of aggression on occupancy increased with competitor density. At low competitor density (25th percentile), increasing aggression from the 25th to the 75th percentile was associated with an increase in occupancy (Δ occupancy_*25th−75th* percentile_ = 0.035 [0.023, 0.046]), whereas at high competitor density (75th percentile) the same increase in aggression was associated with a substantially larger increase in occupancy (Δ occupancy_25th−75th percentile_ = 0.119 [0.099, 0.139]; χ²₁ = 275.69, *P* < 0.001; Fig. 5b).While male occupancy generally increased with female density on the RDA, this effect depended on male aggression. The positive effect of female density on occupancy was stronger for less aggressive vs. more aggressive males (25th percentile: Δ occupancy = 0.0379 [0.028, 0.048] vs. 75th percentile: Δ occupancy = 0.027 [0.017, 0.036]; *X*^2^_1_ = 17.03, *P* < 0.001, Fig. 5c).

### Females

**Fig. 6:**
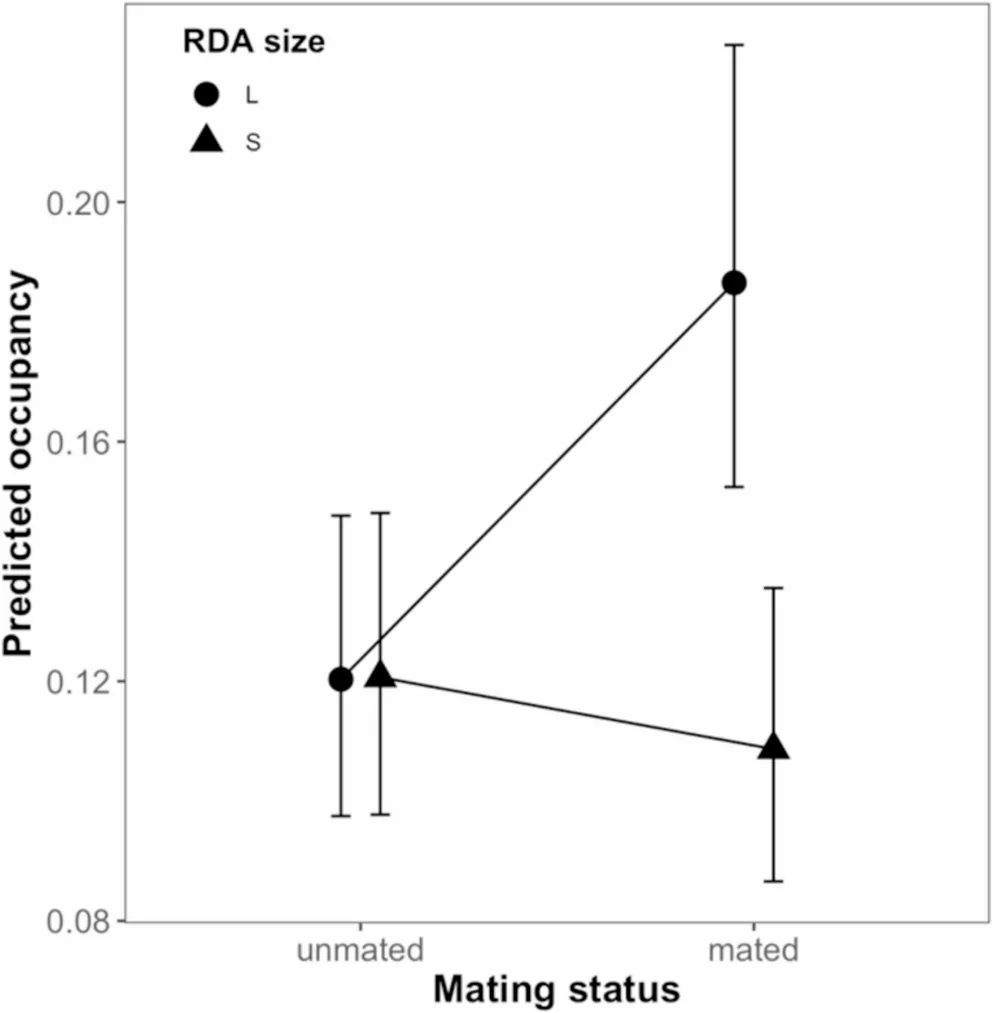
Predicted female occupancy (proportion of frames in RDA; mean ± 95% confidence intervals) of large RDAs increases for mated females compared to unmated females

Here, we tested the prediction that unmated females will spend more time in large RDAs to mate with the most competitive males and avoid those high-density RDAs after mating. In a betabinomial repeated-measures GLMM (with the same structure as described above, *n*_*tot*_ = 16,080 (30 one-minute intervals, 320 individual females, 2 RDAs [across 40 arenas], excluding 2,628 observations due to ongoing matings, and 492 observations excluded by the model due to missing data), females spent significantly more time in high-quality RDAs (Δ occupancy_*low-high*_ = 0.052 [0.036, 0.069], *z* = 7.11, *P* < 0.001; Table S21) and more time in RDAs with a higher number of males present (Δ occupancy_25th-75th percentile_ = 0.031 [0.021, 0.041], *z* = 6.74, *P* < 0.001). This latter finding likely reflects males actively seeking out females (as demonstrated in the male analysis) rather than a primary female preference for areas with numerous males. Conversely, females spent more time on RDAs with a greater number of other females present (Δ occupancy_*25th-75th percentile*_ = 0.024 [0.015, 0.033], *z* = 5.35, *P* < 0.001). We also found a significant interaction of mating status and RDA size on occupancy (*X*^2^_1_ = 49.99, *P* < 0.001, Fig. 6). While unmated females overall spent less time in any RDA (Fig. 4b), mated females showed a significant increase in occupancy-L (Δ occupancy-L_*unmated-mated*_ = 0.066 [0.042, 0.091]). Females remained unmated for a mean ± SD of 16.8 ± 10.2 min of the 30-minute observation period, with 28.4% of females remaining unmated for the entire observation period.

## Discussion

While females allocated approximately 33.2% of their non-mating time to RDAs (including the buffer zone around the small RDA), a substantial 53.2% of mating events started within an RDA or its buffer zone, although mating pairs frequently moved outside these areas before copulations concluded. Consistent with previous research on *D. prolongata*, our results suggest that males spend more non-mating time in RDAs than females, while females visit these areas for copulation (De Nardo et al. 2025a). A higher percentage of mating events commenced in the large (41.6%) compared to the small RDA and its buffer zone (11.7%), despite the lack of a clear preference for large RDAs in unmated females. This observation suggests that females may specifically seek out large RDAs for copulation, potentially due to the presence of highly competitive males. Contrastingly, they may otherwise avoid these areas due to an elevated risk of harassment at high male densities. This interpretation is supported by a mating-associated shift in spatial use: whereas unmated females showed similar occupancy of small (12%) and large RDAs (12.1%), mated females reduced their use of small RDAs (10.9%) while increasing their occupancy of large RDAs (18.7%) by 55%. This may indicate the preferred use of large over small RDAs not only for copulation but also for oviposition.

Studies in *D. melanogaster* show various changes in behavior related to oviposition after mating, including an increase in locomotor activity (Isaac et al. 2010), increased food intake or time spent on food patches (Carvalho et al. 2006; Bath et al. 2017) and preference for yeast in particular (Corrales-Carvajal et al. 2016), as well as increased aggression toward other females on food patches (Bath et al. 2017, 2021). These studies are compatible with our finding that mated females spend more time in areas likely preferred for oviposition. The particular attraction of mated females to large but not small RDAs aligns with a study on *D. melanogaster* demonstrating a preference for oviposition in areas with more aggregated resources compared to those dispersed across multiple smaller sites (Churchill et al. 2021). Small, dispersed resource patches may be perceived as less suitable, for example, potentially due to the limited local larval nutrition, necessitating extensive larval foraging distances (Schwartz et al. 2012), and a higher propensity for desiccation compared to larger patches (Churchill et al. 2021).

Alternatively, if our results do not reflect a preference for larger RDAs as oviposition sites, an increased yeast intake after mating, as reported in *D. melanogaster* (Carvalho et al. 2006; Corrales-Carvajal et al. 2016; Bath et al. 2017), could explain why occupancy of large RDAs increased in mated females. As mating modulates female chemosensory perception and preference (Hussain et al. 2016), this can also make them more sensitive to larger amounts of certain compounds (Scheidler et al. 2015), and may thus attract them to larger aggregations of food.

While the benefits of high-quality oviposition sites may have increased the presence of mated females in large RDAs, unmated females spent considerably less time in RDAs overall. We initially hypothesized that mated females would largely avoid RDAs to evade harassing males. However, while recently mated females may have experienced reduced male attention, unmated females were likely subjected to male harassment (Rowe et al. 1994), particularly in RDAs with high densities of competing males. Previous research has demonstrated that male harassment can significantly alter female oviposition and foraging patterns, often to the detriment of female fitness (Stone 1995; Bauer et al. 2005; Darden and Croft 2008; Bacon and Barbosa 2020), thus providing a plausible explanation for the low occupancy of beneficial resources by unmated females. Nonetheless, considering that many matings started within RDAs indicates that unmated females do seek out such areas for copulation, as predicted, although this is not reflected in their general occupancy rates. As discussed in the introduction, mating with territorial males may reduce mate choice costs (Candolin and Voigt 2001; Deb and Balakrishnan 2014) and could indirectly lead to selection of high-quality mates (Kokko et al. 1999; Castellano 2009; Kovalov and Kokko 2022), resulting in indirect fitness benefits (Candolin and Voigt 2001; Seddon et al. 2004; Vanpé et al. 2007; White and Rundle 2015). While a substantial proportion of females mated in RDAs, a significant percentage did not, suggesting that females may adopt different mating strategies depending on their individual condition. This is further explored in a second study on the same experiment (De Nardo et al. 2026).

Similar to females, males spent more time in large (24.4%) compared to small RDAs (11.1%, including buffer). Analogous to the potential reasons for female preference, males may perceive larger resource aggregations as more valuable and worth defending, because they are preferred as oviposition sites by females (Hoffmann and Cacoyianni 1990). Consistently, *D. melanogaster* males aggregate more readily on clustered resources forming a single large area rather than several dispersed patches (Churchill et al. 2020), mirroring female oviposition preferences on such clustered resources (Churchill et al. 2021). However, the male preference for occupying large RDAs comes with significant costs: occupancy of large RDAs is predicted to a much greater extent by an individual’s aggression rate compared to small RDAs, likely incurring substantial energetic costs (Hoffmann and Cacoyianni 1989; Guo and Dukas 2020) and an increased risk of injury (e.g., Geist 1986; Piper et al. 2008). Despite these high costs, males achieved exclusive occupancy of large and high-quality RDAs less frequently than of small or low-quality RDAs, indicating lower defensibility. Yet the prevalence of males accessing and defending large RDAs implies that significant fitness benefits can be derived from territoriality in these locations. De Nardo et al. (2026), based on the same experiment, confirms that occupancy of the large RDA increases mating success in large, but not small males, which use a scramble competition tactic to secure mating opportunities.

Interestingly, when more females were in an RDA, males exhibiting low aggression had higher occupancy than in the presence of fewer females. This was unexpected, as the intensity of competition often increases in the presence of females due to higher incentives (López and Martín 2002; Yuan et al. 2014). However, the influx of females into an RDA alters the local operational sex ratio. Our analysis indicates that both small and large RDAs exhibit a male-biased local OSR on average and throughout the experimental period (Fig. S11). However, with an increased presence of females, the focus of male behavior may shift from aggression toward courtship, and the less male-biased OSR (i.e., closer to 1:1) may attenuate the intensity of male-male competition (Grant and Foam 2002; Weir et al. 2011; De Nardo et al. 2025a). This observation also aligns with the largely even OSR in the small RDA compared to the more male-biased OSR in the large RDA, corresponding to the relative aggression rates needed for occupancy in each area. Alternatively, instead of a shift in behavior from aggression to courtship by the males that are present, an increase in females may attract additional males (e.g., scrambling males) to enter an RDA, that are on average less aggressive than those that are occupying the RDAs for longer periods.

In conclusion, this study provides new insight into how the spatial distribution of resources can significantly influence the movement and behavioral patterns shown by *D. prolongata* males and females and how these resources are used by individuals depending on the levels of competition, their individual status and how much effort they can invest in the defense of resources. While both sexes demonstrated a preference for larger and higher-quality RDAs, the underlying reproductive tactics driving these preferences differed considerably. Males prioritized the occupancy and defense of these attractive sites, accepting higher levels of competition and associated costs to maintain access to females. Conversely, female behavior appeared to be status-dependent, transitioning from unmated avoidance of large RDAs to post-mating preference for them for feeding or oviposition. Our findings underscore that resource distribution not only shapes individual behavior but also interacts in complex ways with the behavior of conspecifics of both sexes, an individuals’ own state, and the environmental context to affect movement patterns, local sex ratios, the efficacy of territorial contest, and the coexistence of reproductive tactics in a population. To gain a more comprehensive understanding of the complexity inherent in mating systems and reproductive tactics across sexes, it is crucial to incorporate environmental factors such as the heterogeneous distribution of resources, as this can drastically alter the ecological and evolutionary dynamics within a population.

## Supplementary Information

Below is the link to the electronic supplementary material.

Supplementary File 1 (PDF 8.12 MB)

## Data Availability

The data is provided within the supplementary information files.
