# Supplementary material for: Sex-specific site preferences in response to resource value in Drosophila prolongata
Source: Behav Ecol Sociobiol. 2026 May 8;80(5):63. doi: 10.1007/s00265-026-03731-7 (PMC13654897; doi:10.1007/s00265-026-03731-7)
Supplement: Supplementary file 1 [file 265_2026_3731_MOESM1_ESM.pdf]

Supplementary Information

for

"Sex-specific site preferences in response to resouce value in *Drosophila prolongata*"

Swarnima Mukherjee, Alessio N. De Nardo & Stefan Lüpold

Supplementary Information

Figures

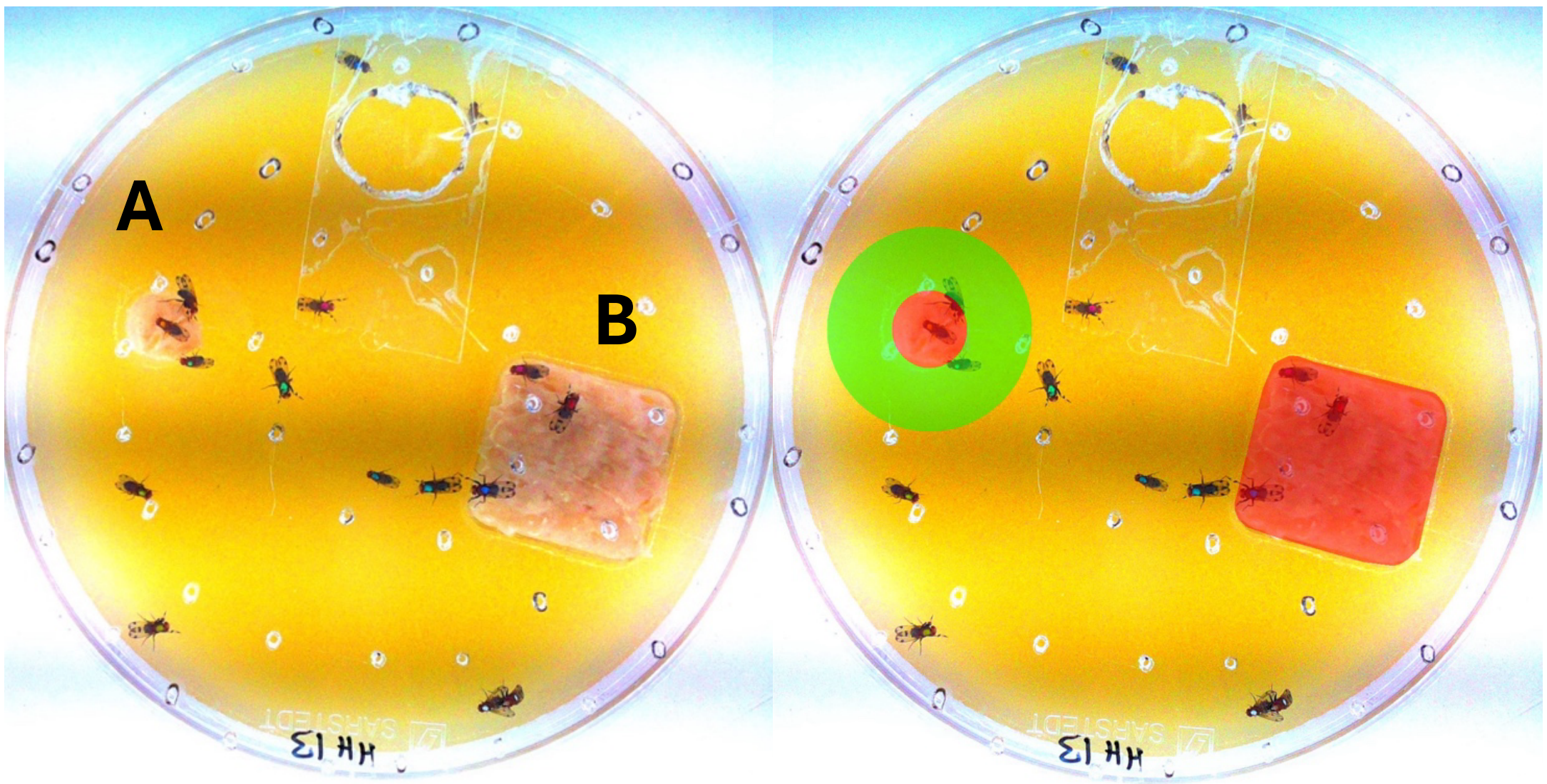

Figure S1: The left image shows an example of an experimental unit, in this case with a small (A) and large (B) resource-dense area (RDA) of high quality. The right image shows the same experimental unit, with the regions of interest (ROIs) overlayed on the RDAs. The red areas mark the RDAs, whereas the green area marks the buffer zone added around the small RDA, to enhance comparability of their occupancy.

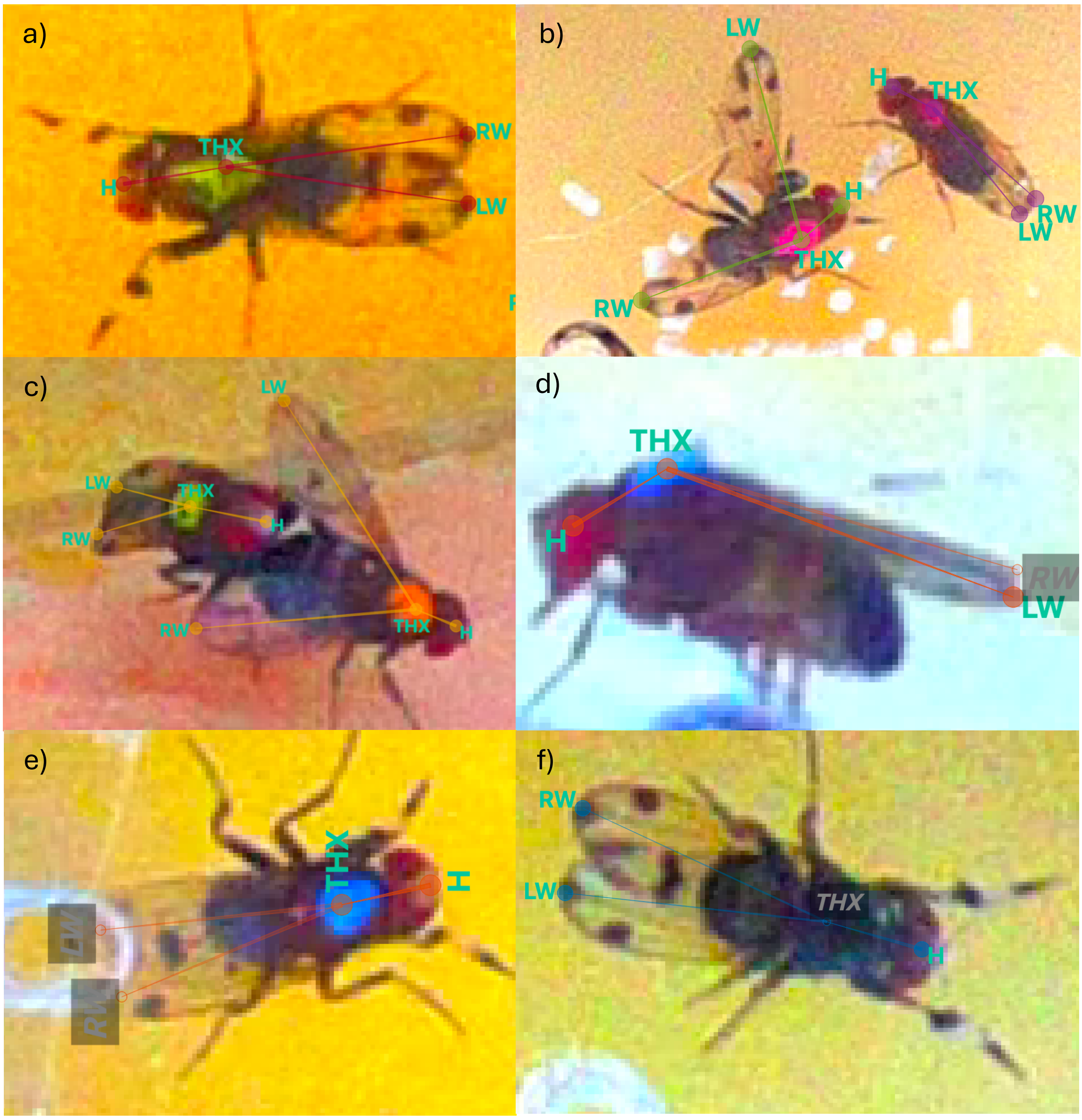

Figure S2: Examples of labeled instances in various postures to train the SLEAP model: a) standard sitting position, skeleton in color, body parts in green (H = head, THX = thorax, LW = left wing, RW = right wing); b) male courting female; c) mating pair; d) female sitting on the edge from the side, invisible body part in grey; e) male with wings partly concealed by a ventilation hole, wings labeled as not visible; f) male sitting on the lid, seen from below, thorax labeled as invisible.

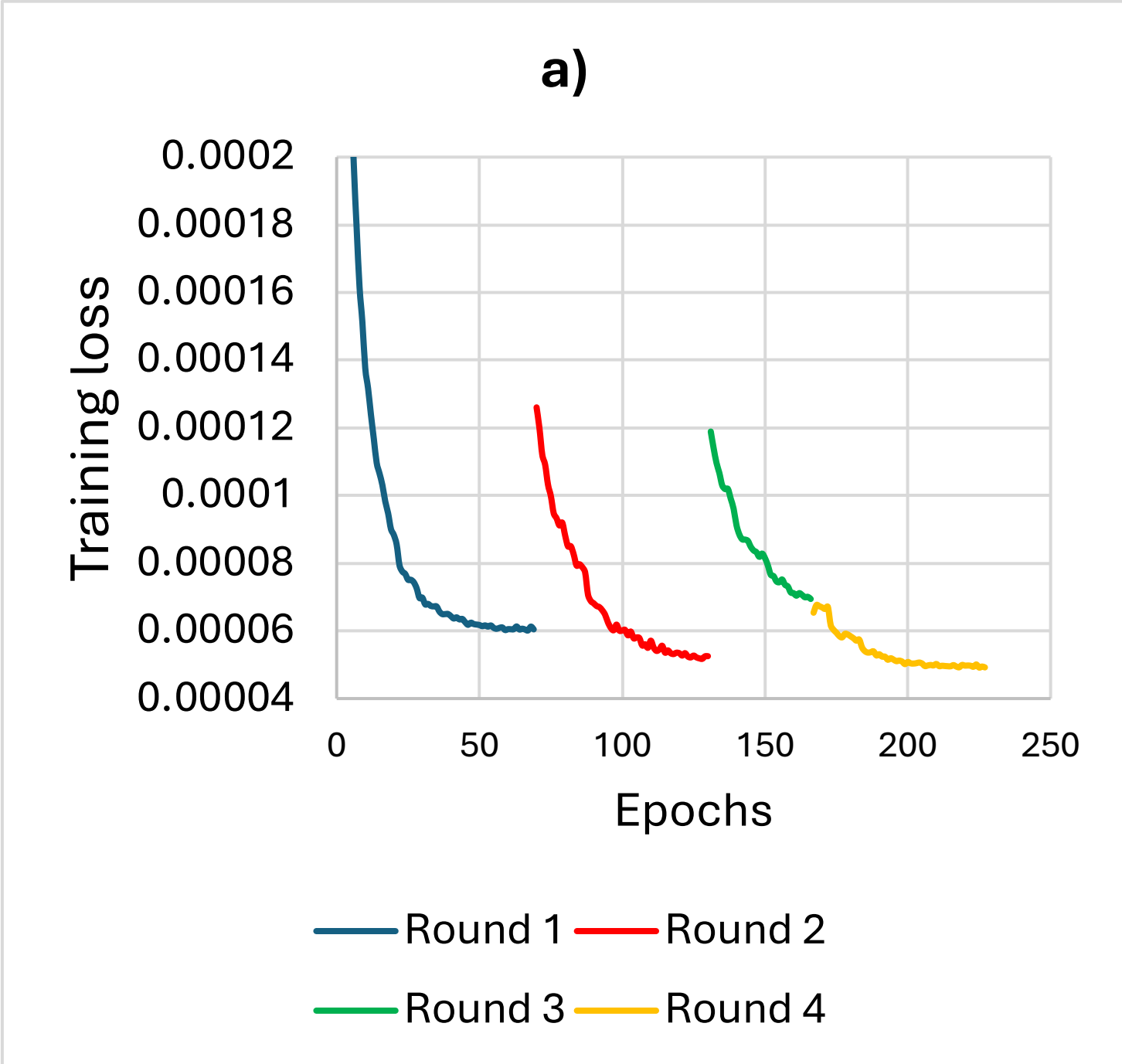

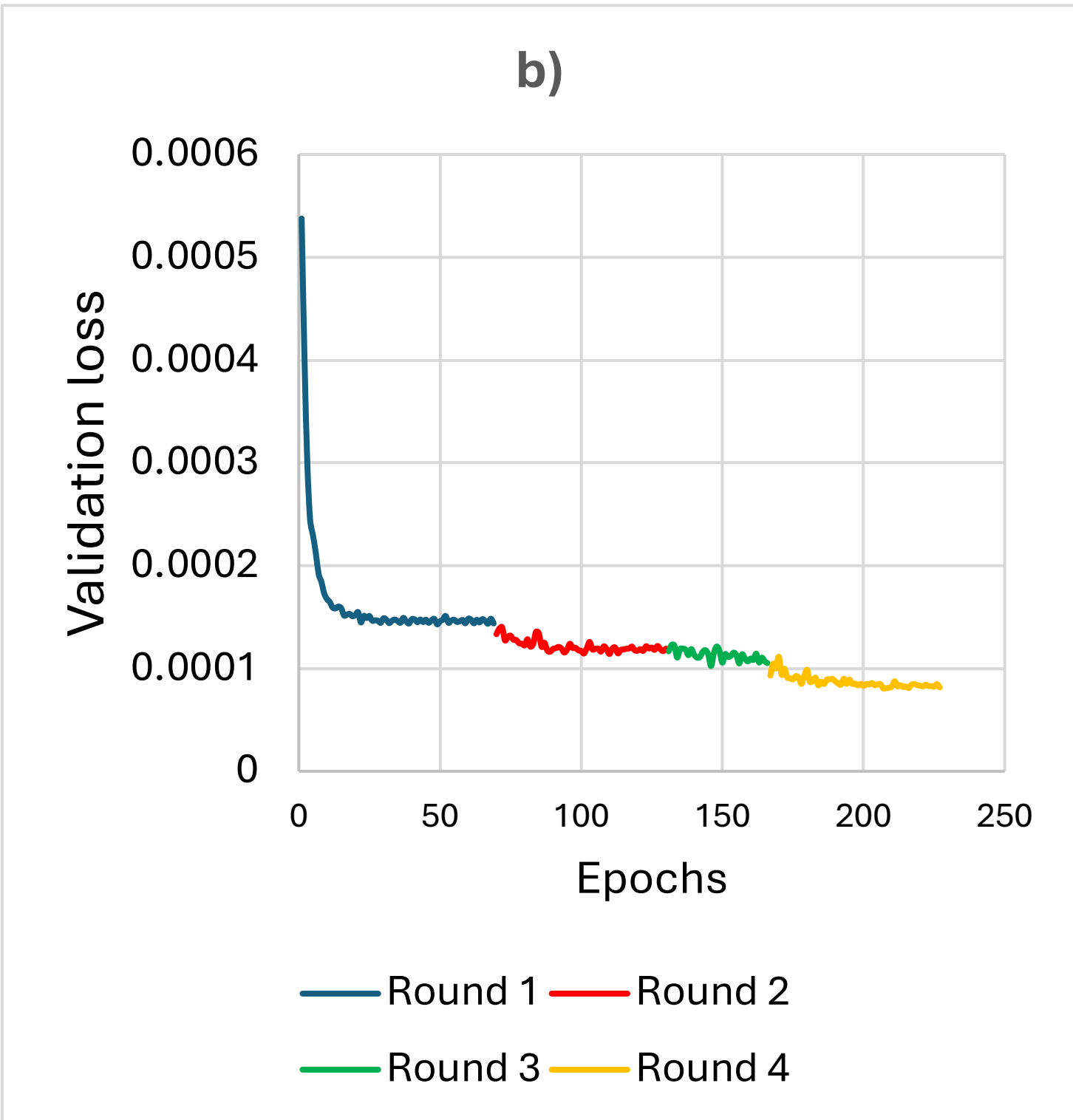

Figure S3: Loss curves showing reduction of the error (y-axis) between model predictions and ground truth (i.e., provided validation labels) across training epochs and rounds on **a)** the training set and **b)** the validation set. An epoch is a full pass through a training set (i.e., all training frames are learned once per epoch). Rounds of training are additional training sessions (adding more epochs of total training) but after adding additional training data. Loss curves on training data show how the model improves on the data it has seen, however, as new data is added in each round, there is an uptick before there is a reduction. The more important value is the validation loss, showing how prediction on unseen data improves.

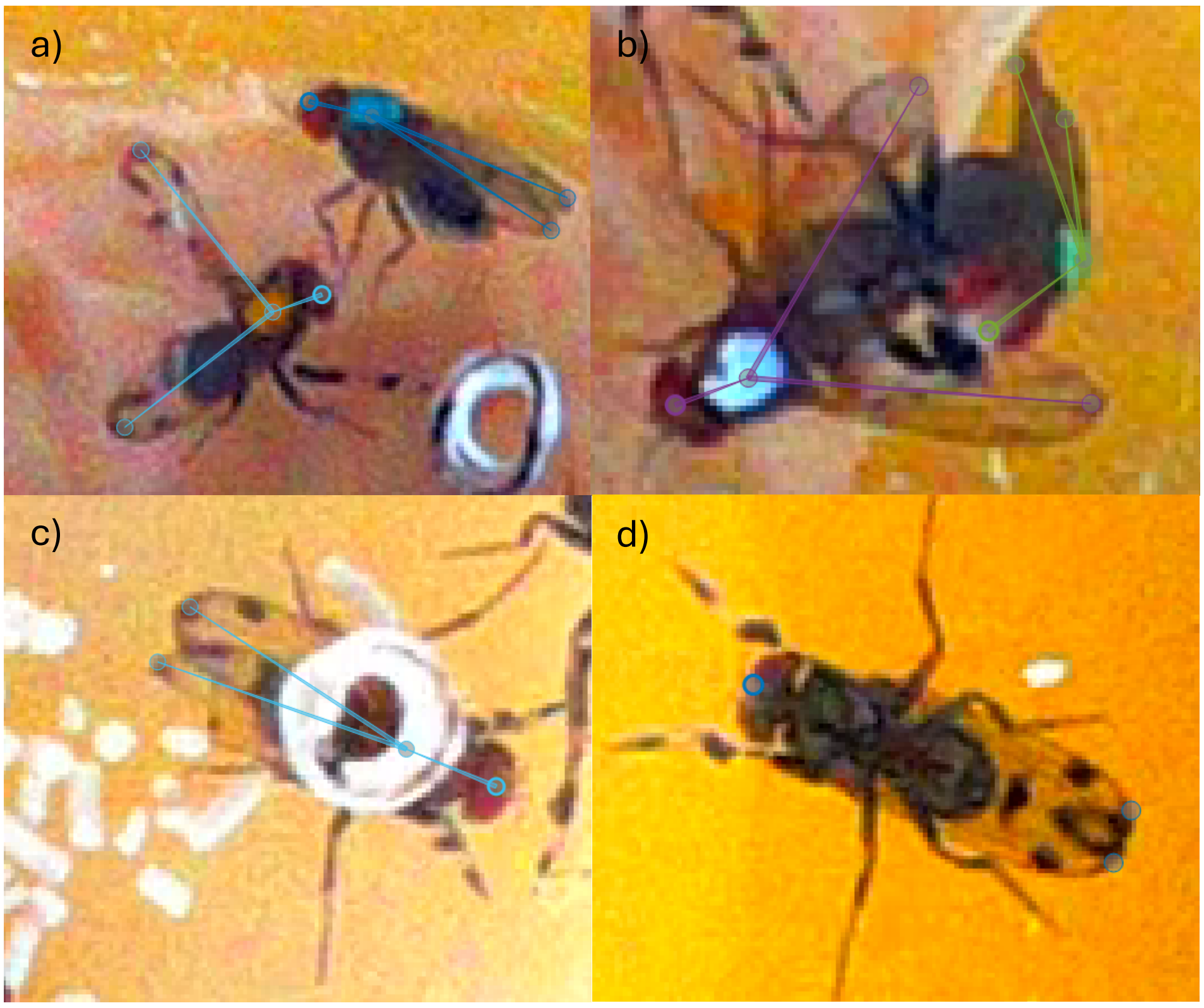

Figure S4: Examples of instances predicted by our trained SLEAP model: the model does well on **a)** a male courting a female, **b)** a mating pair **c)** a male sitting below a ventilation hole (even invisible thorax still correctly predicted), and **d)** a male sitting on the lid from below. Note that the model correctly does not predict the thorax.

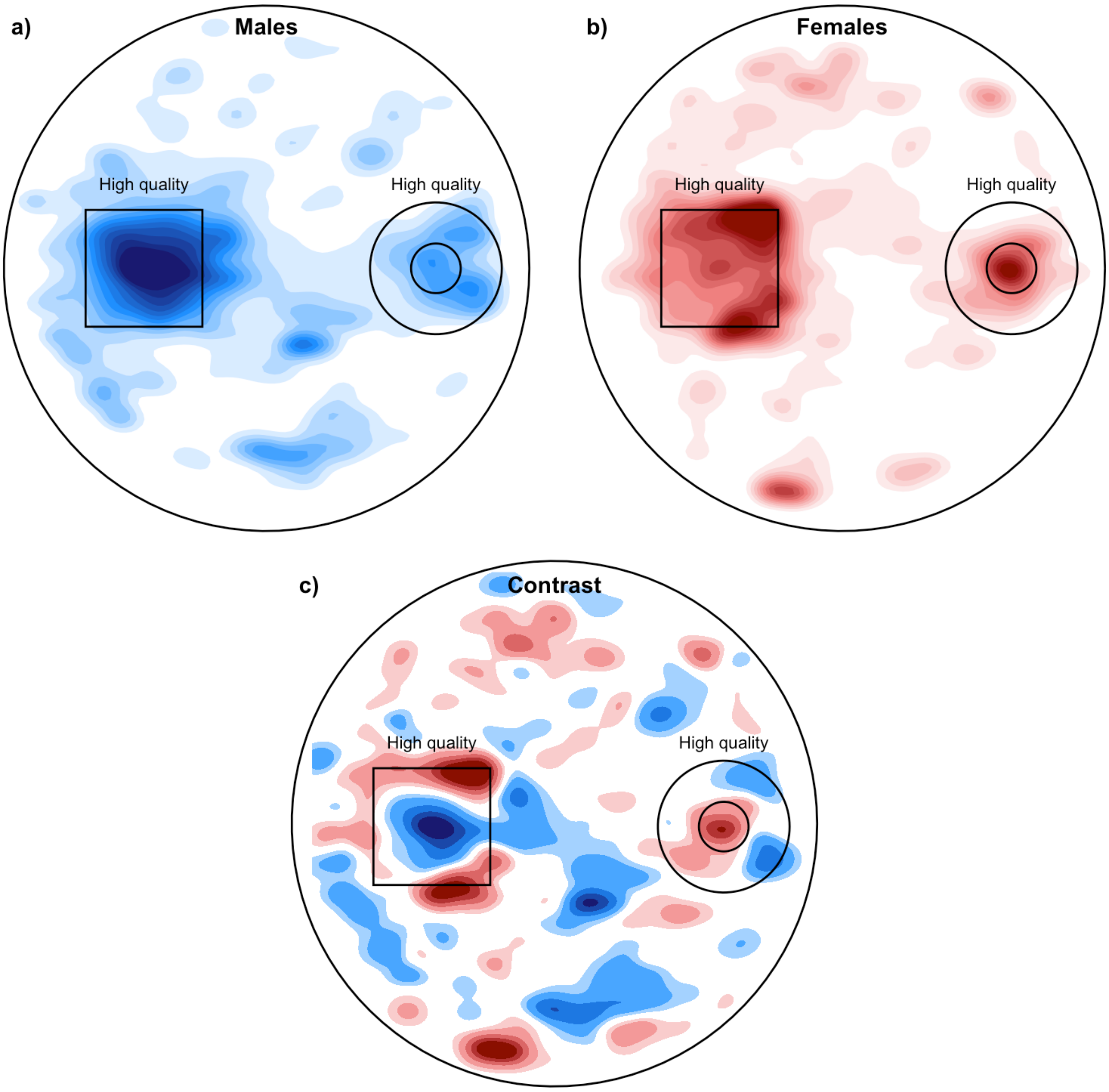

Figure S5 (part 1/4): Distribution of a) males and b) females around two high-quality RDAs, with the large RDA outlined by the square and the small RDA and its buffer outline by the two concentric circles. Darker colors indicate higher density of flies across all frames and units. Panel c) shows the contrast of male and female densities, with blue colors showing areas where males were more frequently observed and red areas showing areas where females were more frequently observed. White areas indicate similar male and female density.

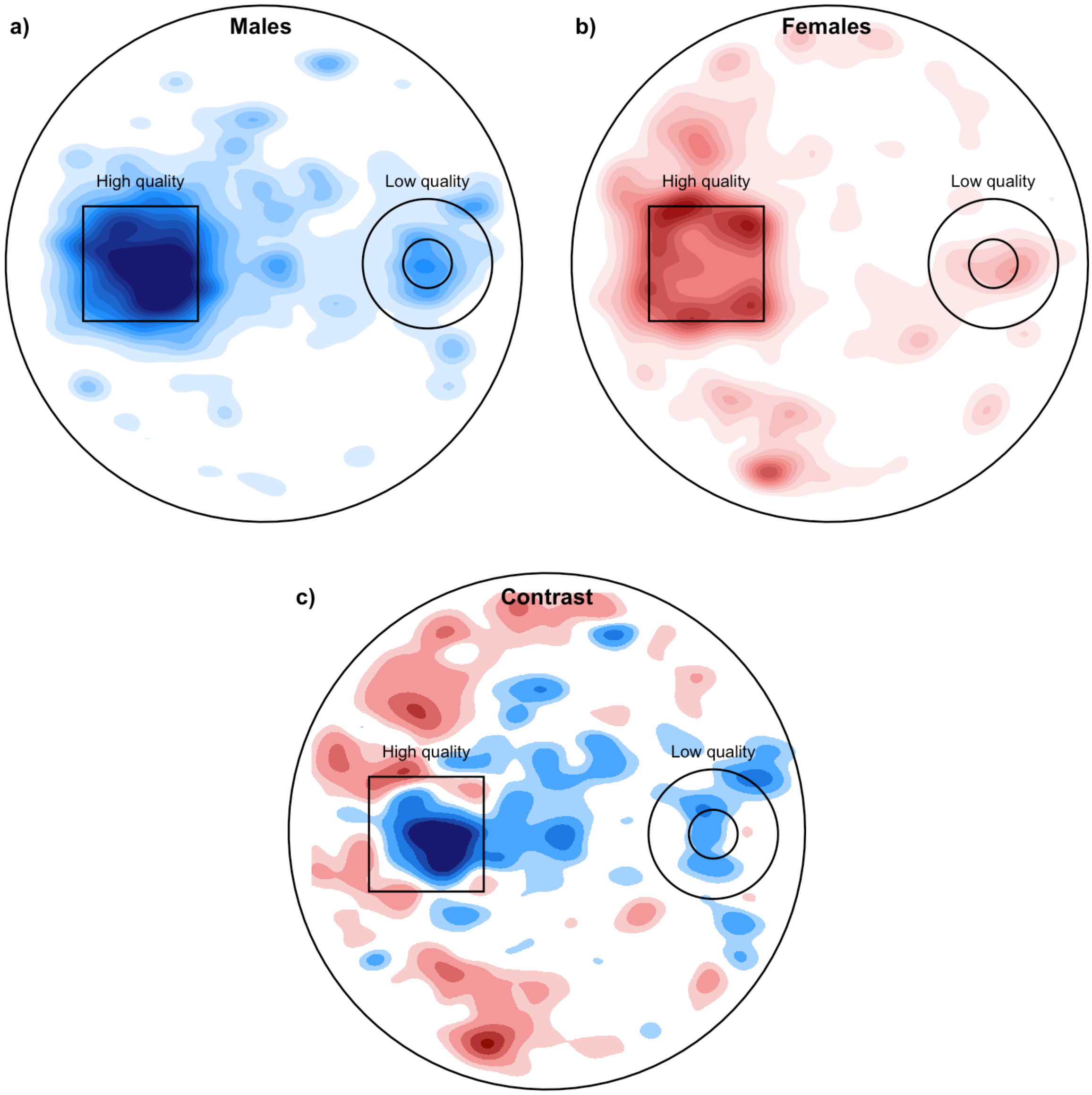

Figure S5 (part 2/4): Distribution of a) males and b) females around a large high-quality RDA and a small low-quality RDA, with the large RDA outlined by the square and the small RDA and its buffer outline by the two concentric circles. Darker colors indicate higher density of flies across all frames and units. Panel c) shows the contrast of male and female densities, with blue colors showing areas where males were more frequently observed and red areas showing areas where females were more frequently observed. White areas indicate similar male and female density.

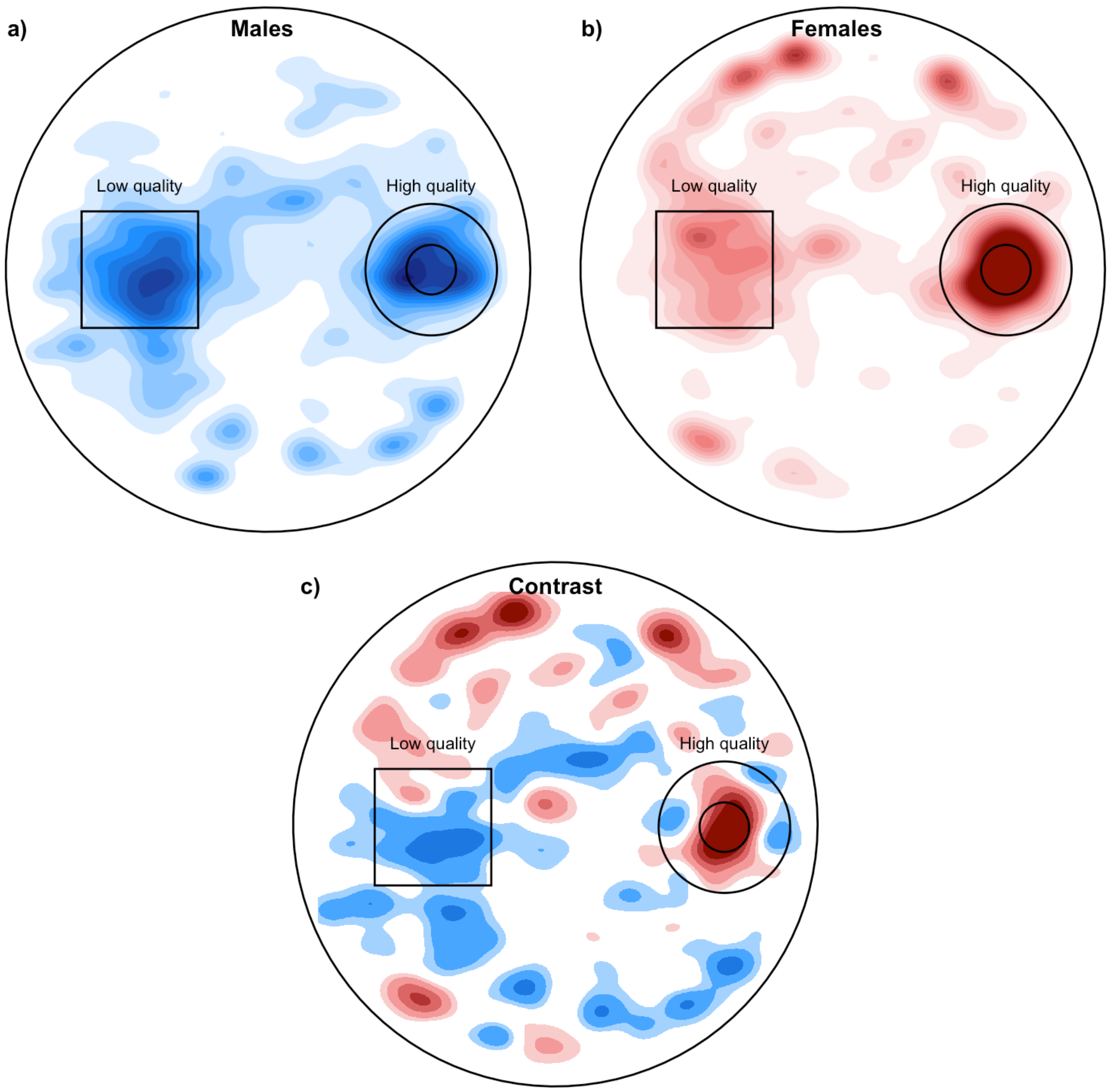

Figure S5 (part 3/4): Distribution of a) males and b) females around a large low-quality RDA and a small high-quality RDA, with the large RDA outlined by the square and the small RDA and its buffer outline by the two concentric circles. Darker colors indicate higher density of flies across all frames and units. Panel c) shows the contrast of male and female densities, with blue colors showing areas where males were more frequently observed and red areas showing areas where females were more frequently observed. White areas indicate similar male and female density.

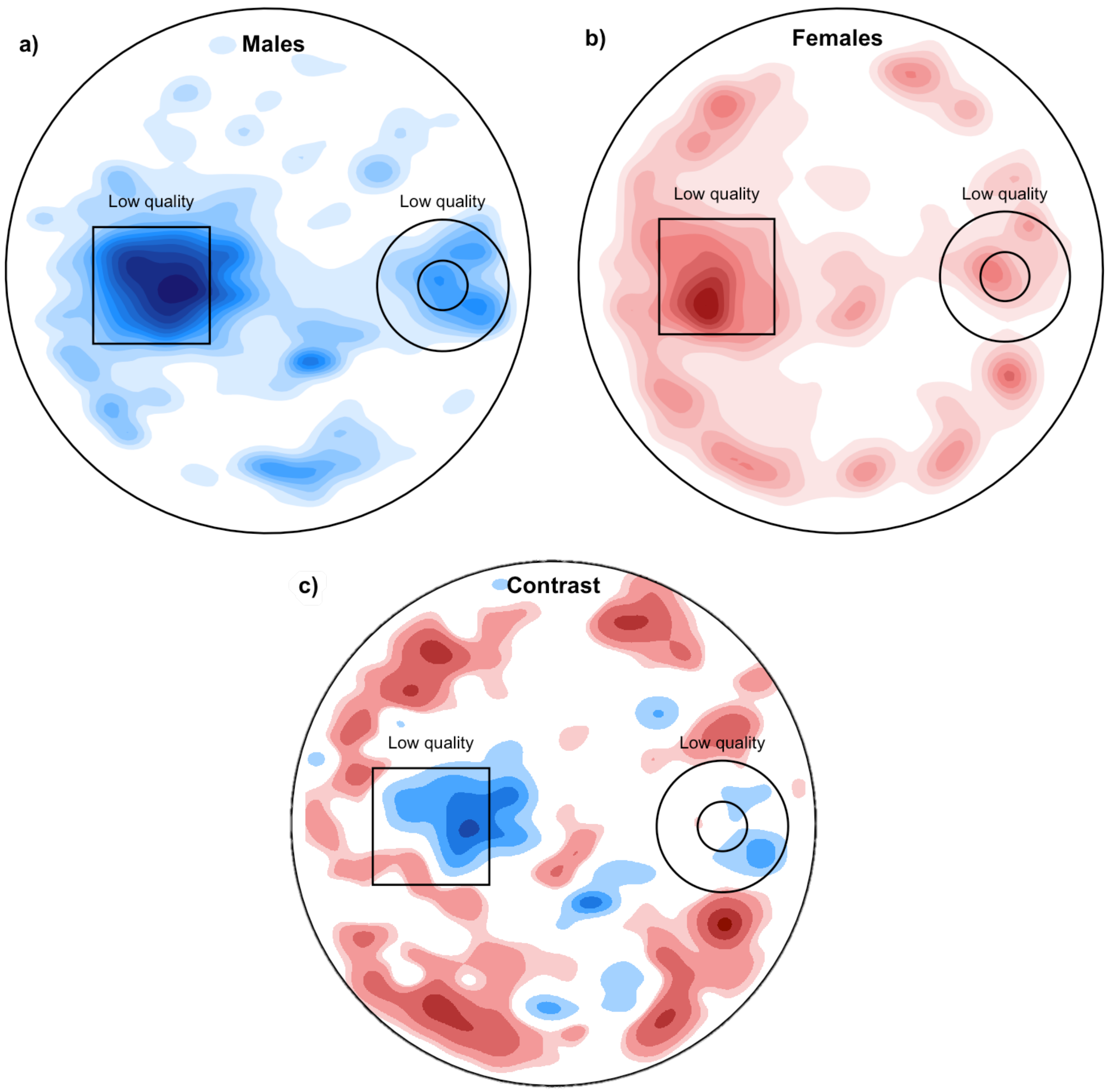

Figure S5 (part 4/4): Distribution of a) males and b) females around two low-quality RDAs, with the large RDA outlined by the square and the small RDA and its buffer outline by the two concentric circles. Darker colors indicate higher density of flies across all frames and units. Panel c) shows the contrast of male and female densities, with blue colors showing areas where males were more frequently observed and red areas showing areas where females were more frequently observed. White areas indicate similar male and female density.

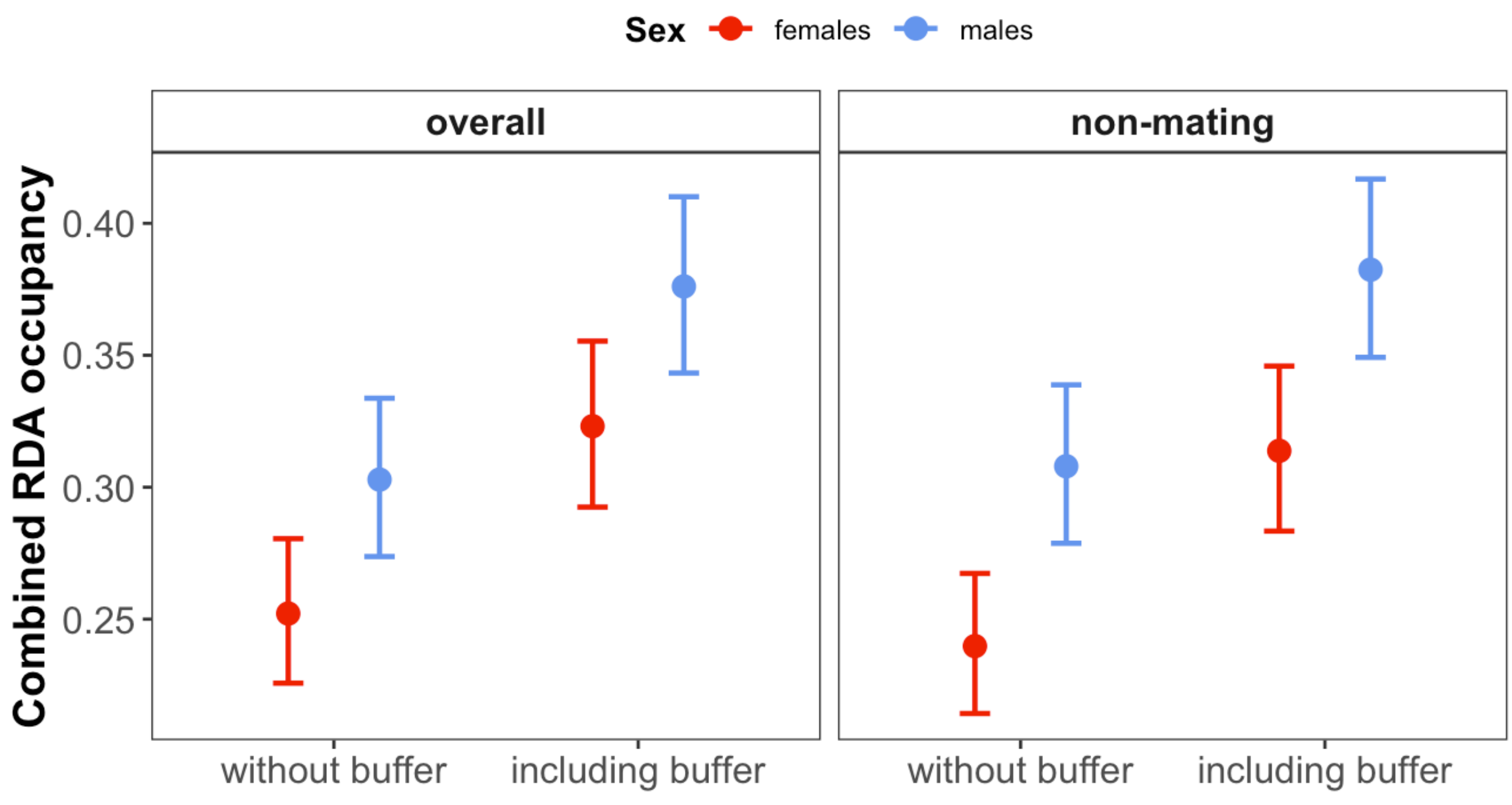

Figure S6: Combined (small and large) RDA occupancy (proportion of frames in RDA; mean ± 95% confidence intervals), depending on sex, inclusion of the buffer zone, and whether occupancy is considered during copulation or not.

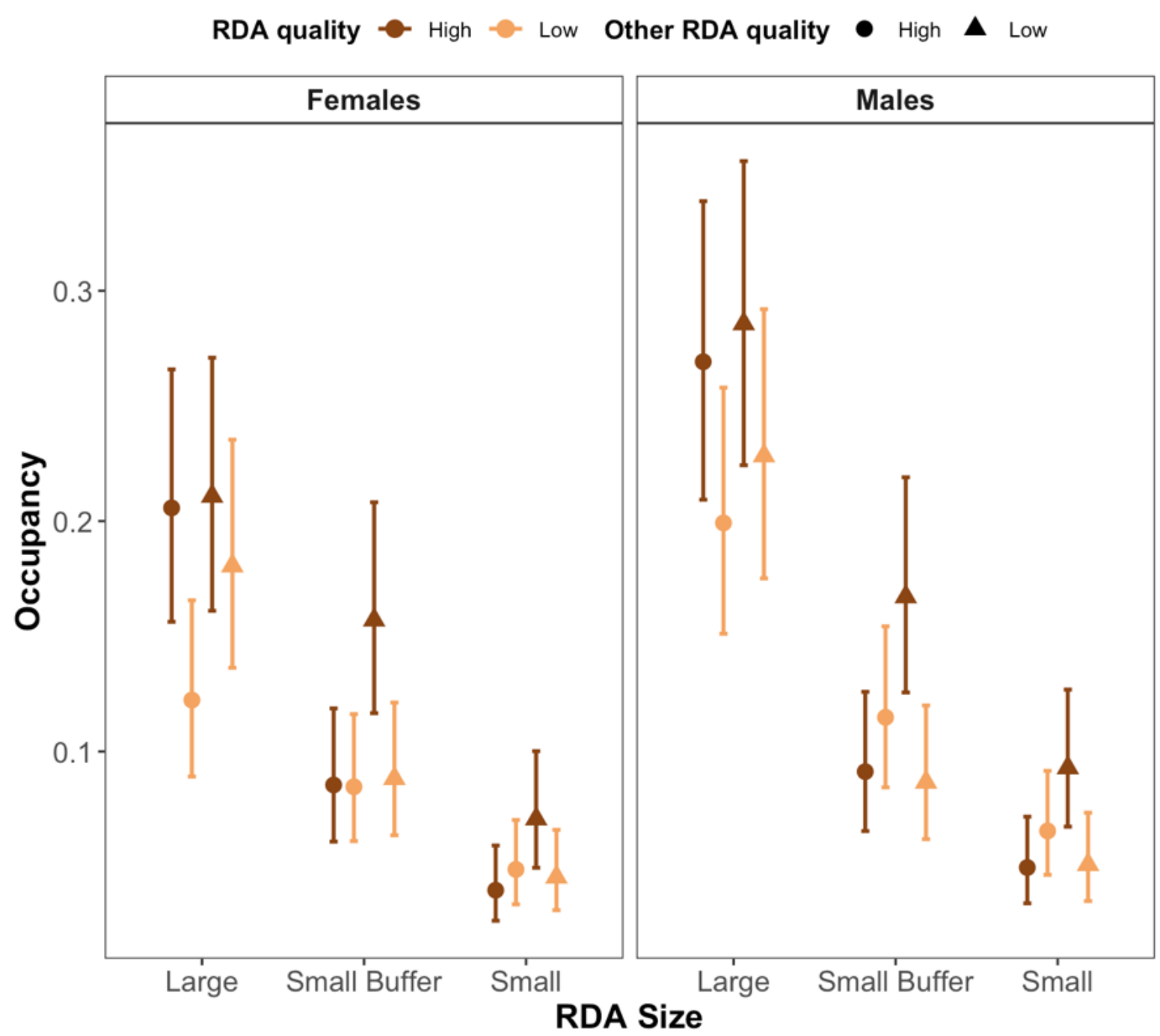

Figure S7: Occupancy (proportion of frames in RDA; mean ± 95% confidence intervals) of resource-dense areas (or their buffer zone), depending on sex, quality, and quality of the alternative RDA.

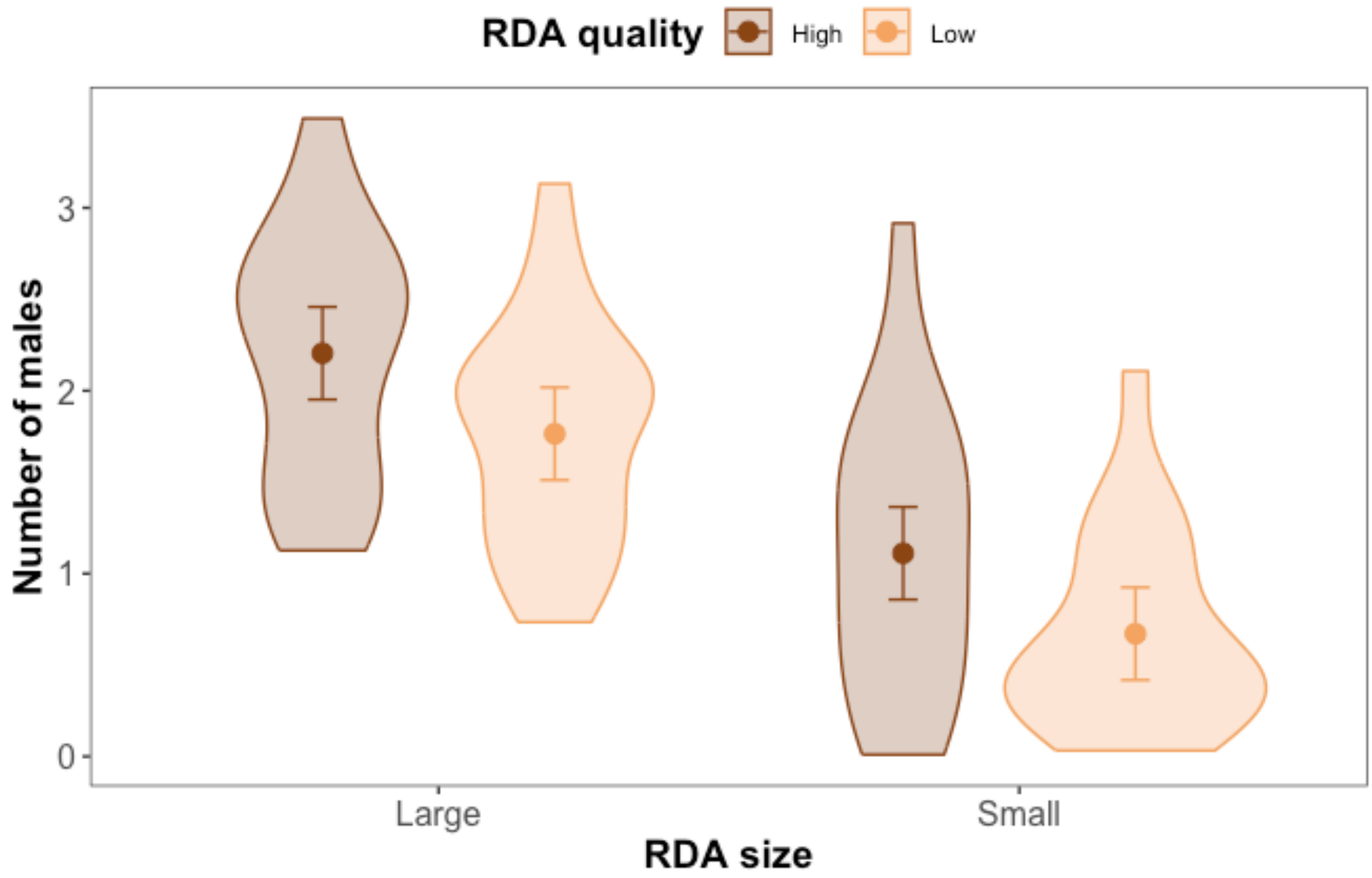

Figure S8: Average number of males on RDA (mean ± 95% confidence intervals) depending on RDA size and quality

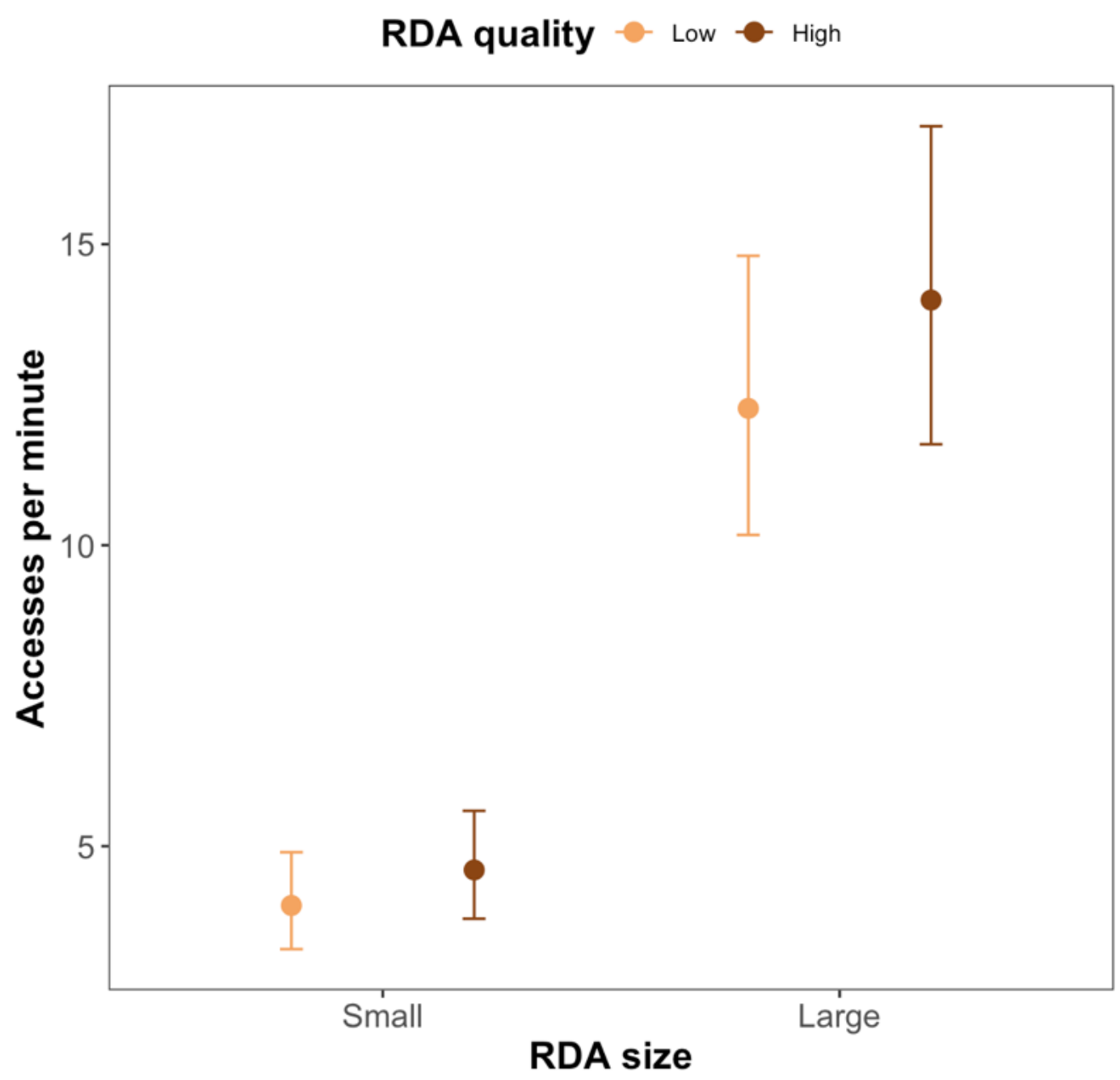

Figure S9: Total RDA accesses per male (mean ± 95% confidence intervals) depended on RDA size but not quality

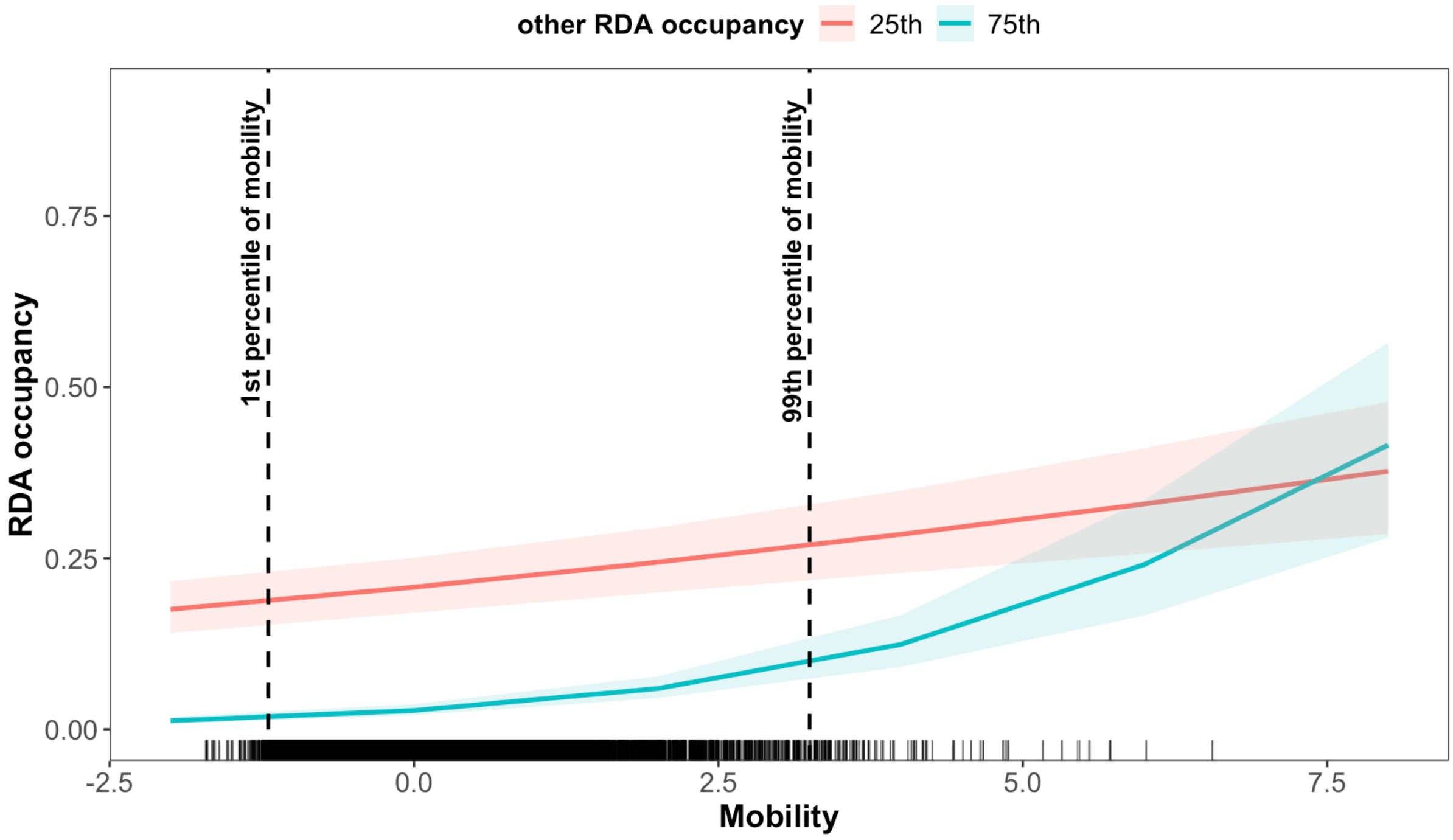

Figure S10: The interaction effect of mobility and occupancy of the other RDA on male focal RDA occupancy is statistically significant, yet biologically irrelevant as it is only visible at a mobility higher than the 99th percentile. The ticks on the x-axis show the distribution of data points of mobility. Whilst more mobile males show slightly higher occupancy regardless of the occupancy of the other RDA, this effect is the same at the 1st and 99th percentile of mobility.

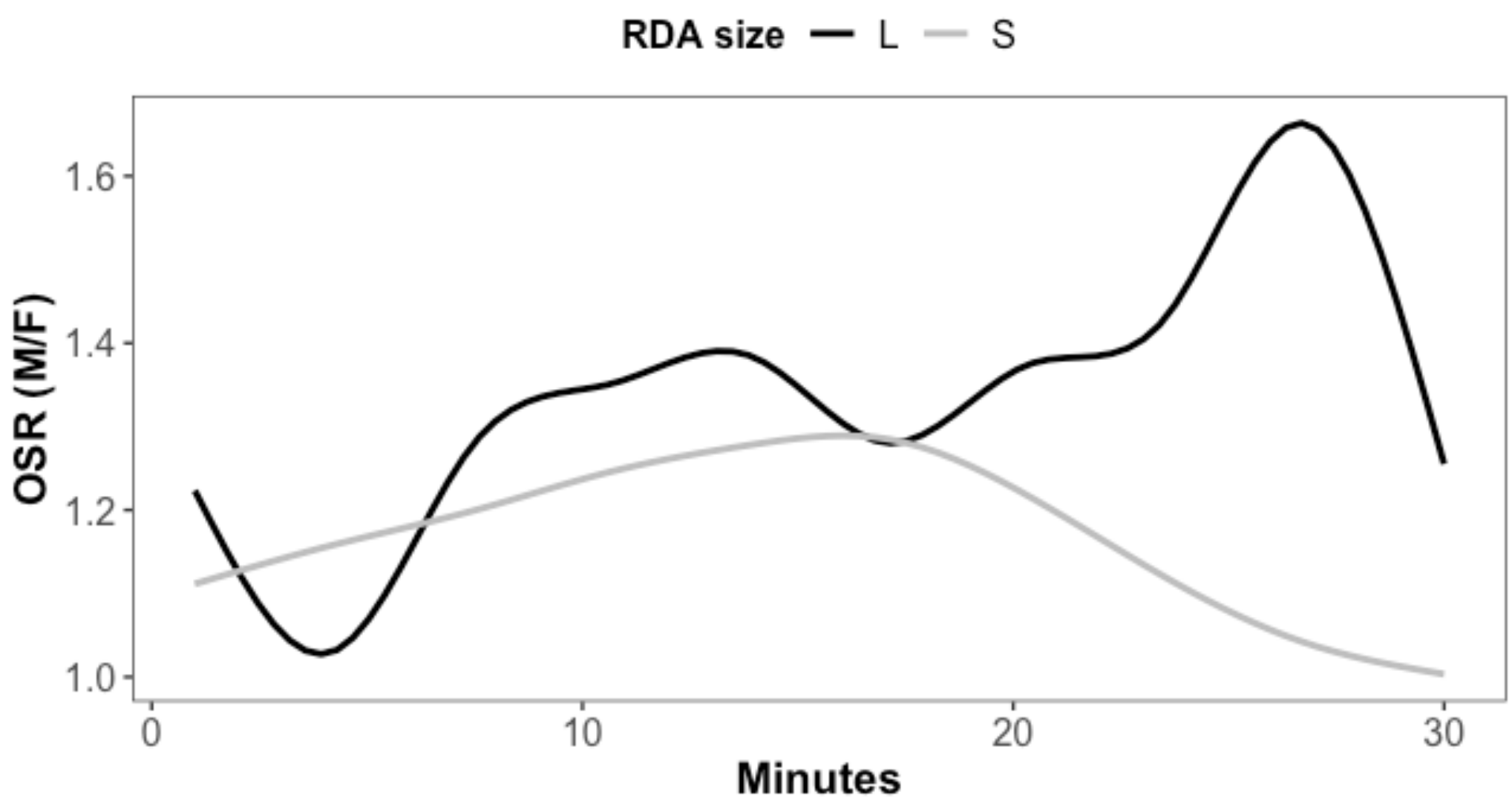

Figure S11: Local operational sex ratio (OSR) in RDAs depending on their size over time. OSR was calculated as the number of males divided by the number of females. Values above 1 indicate a male bias, whereas values below 1 indicate a female bias.

Supplementary Tables

Table S1: Overview of sample sizes for training the SLEAP model.

Round	*Number of new frames*	*Number of total frames*	*Number of total instances*	*Validation set*
1	160<br>(20 frames across 8 units = 2 units per treatment)	160	2,560	160<br>(20 frames across 8 units = 2 units per treatment)
2	320<br>(40 frames across 8 units)	480	7,680	160
3	480<br>(60 frames across 8 units)	960	15,360	160
4	640<br>(80 frames across 8 units)	1600	25,600	160

Table S2 (part 1/2, continued next page): Overview of training parameters for the centroid model and the centered instance model in SLEAP

Parameter	*Explanation of parameter*	*Centroid Model*	*Centered Instance Model*	*Explanation of values*
Input Scaling	Scale factor for input images	0.5	1	Adjusts the size of input images, affecting processing speed and potentially accuracy. As the centroid model only localizes the area containing instances, but has much lower processing speed, input scaling is reduced for that model
Batch size	Number of images processed together	4	4	Larger numbers increase computational load, but also increase generalizability
Epochs	Maximum number of times the entire dataset is processed, may be stopped earlier depending on plateaus	200	200	Total rounds of training over complete dataset. Maximum is 200 but never reached, because always stopped early. See plateau and early stopping
Optimizer	Algorithm for optimizing network	Adam	Adam	Popular optimization algorithm known for efficiency and effective convergence
Initial learning rate	Starting learning rate for optimization	0.0001	0.0001	Sets the step size at the beginning of training, crucial for convergence. A high learning rate would fast convergence, but may overshoot, low learning rate could prevent overshooting but risk of getting stuck in local minimum. However, here the learning rate is not constant and are adapted at stagnation.
Reduce on plateau	Whether to reduce learning rate on plateau	Yes	Yes	Reduces learning rate when plateau is reached (no progress for specified number of epochs), to help fine tune model performance
Plateau patience (learning rate)	Number of epochs to wait before reducing rate	5	5	After 5 epochs without progress, the learning rate will be reduced

Table S2 (part 2/2)

Early stopping	Whether training can stop early	Yes	Yes	Training can stop early, if no improvement after
Plateau patience (early stopping)	Patience for early stopping	20	10	Depending on the model, training is stopped if no improvement for 20 or 10 epochs
	Neural network architecture used for processing	U-Net	U-Net	U-Net: a type of convolutional network known for effectiveness (Ronneberger et al. 2015)
Max Stride	Maximum downsampling factor in the network	16	16	The network reduces the input images by up to a factor of 16 at its deepest layer
Output Stride	Downsampling factor for output relative to input	2	4	Resolution of output relative to input
Filters	Initial number of filters in the network	16	24	Filters enable the network to extract more features, affecting the possible complexity of what can be learned
Filters rate	Rate by which filter number increases form layer to layer, allows to process increasingly complex features	2	2	A filter rate of 2 means the number of filters is doubled in each layer
Up interpolate	Whether to use upsampling in the network	Yes	Yes	Enables the process of increasing resolution of feature maps that were reduced during downsampling and can improve precision
Anchor part	Reference body part for localization	Thorax	Thorax	Used as a reference point for localization within the model
Sigma	Spread of the peak in the output probability map	2.5	2.5	Influences precision of localization in pixels: smaller values are more precise, but difficult to learn
Augmentation	Various settings can be used for augmentation. The images are altered so that overfitting is avoided, and more robust and generalizable features are learned	**Rotation range:**<br>-180° to 180°<br>**Random flip:**<br>Yes	**Rotation range:**<br>-180° to 180°<br>**Random flip:**<br>Yes	Rotation and random flip allow the images to be rotated or vertically/horizontally flipped, to learn the images in different orientations

Table S3: Precision (proportion of detected flies that really are that body part) and recall (proportion of the total of that body part that were detected) for different body parts, across two haphazardly selected replicates of each RDA quality treatment and two haphazardly selected frames each, with a maximum of 16 detectable individuals per frame ($n = 2 \times 4 \times 2 \times 16 = 256$).

	**Whole fly**	**Head**	**Thorax**	**Wings**
**Precision**	1.0	1.0	0.996	0.975
**Recall**	0.992	0.996	0.995	0.988

Table S4: Overview of settings for tracking instances across frames in SLEAP.

Parameter	*Setting*	*Explanation*
Tracker Method	Flow	Uses optical flow for tracking, i.e., predict movement based on apparent motion
Target Number of instances per frame	No	No fixed number of instances. Set to know to avoid identification of non-target objects as instances if some targets are not visible
Similarity method	Centroid	Uses the centroid (geometric center of objects) as the position of an instance for tracking across frames
Matching method	Hungarian	Combinatorial optimization algorithm that tries to minimize the total cost (i.e., distance) for movements between frames. Tries to find a parsimonious solution.
Elapsed frame windows	10	Allows the Kalman Filter to use the last 10 frames to predict the positions of instances in the current frame. Helps to smooth out trajectory estimates and improves robustness
Robust quantile of similarity scores	0.95	Uses the 95th percentile of similarity scores as a robust threshold to filter matches
Connect single track breaks	yes	Attempts to reconnect

Table S5 (part 1/2, continued on next page): Overview of python pipeline for processing predicted coordinates from SLEAP, before using trained models to predict behaviors

	Python file	*Content*	*Libraries*
1	extract_coord.py	1) Prompts for input directory path, locates .h5 file that contains coordinates exported from SLEAP<br>2) Extracts locations and track names<br>3) Loads the excel file mapping the track names from SLEAP (numeric) to custom track names according to sex and color of the fly<br>4) Separates location data into separate variables for head, thorax, left wing and right wing<br>5) Creates a plot for thorax location tracks, saves it to directory<br>6) Saves the exported coordinate data as coordinates.xlsx in directory	os, numpy, pandas, h5py, matplotlib.pyplot
2	interpolate.py	1) Prompts to enter input directory path, locates coordinates.xlsx file<br>2) Creates a copy of the input file (coordinates_OG.xlsx)<br>3) Missing thorax coordinates are interpolated based on existing wing and head coordinates, if available<br>4) Further missing thorax coordinates are filled in using linear interpolation, using the coordinates in frames that come before and after<br>5) For each individual a variable is added to indicate whether the coordinates have been interpolated or not	os, pandas, numpy, matplotlib.pyplot
3	define_ROIs.py	1) Prompts to enter input directory, locates RoiSet.zip file, which was labeled in Fiji (Schindelin et al. 2012) for each unit<br>2) Extracts ROI (region of interest) definitions for large, small and small buffer ROI from the file<br>3) Stores the x and y coordinates of each pixel belonging to a ROI in a data frame, saves it to roi_pixel_coordinates.xlsx in the directory<br>4) Loads images from the directory (first and last frame of the recording), and creates a copy on which the ROIs are overlayed (for visual check; e.g., Fig. S5), saves them in the directory. The first and last image are used to make sure the RDA remains in the same place throughout the recording (i.e., neither the camera nor the petri dish moved)	os, cv2, numpy, pandas, read_roi, shapely.geometry
4	territory.py	1) Prompts directory and loads coordinates.xlsx and roi_pixel_coordinates.xlsx files<br>2) Identifies individuals depending on their prefix in the variable name<br>3) Check coordinates for each body part for each individual whether they fall within each of the ROIs (1 yes, 0 no), saves them in new variables<br>4) Returns updated coordinates.xlsx	os, pandas
5	wing_angles.py	1) Prompts directory and loads coordinates.xlsx<br>2) Computes wing angle for each individual:<br>- calculates vector from head to each of the wings<br>- computes the angle between these two vectors<br>- stores wing angles for each individual in new columns<br>3) computes wing angle "velocities" for each individual:<br>- the change in wing angle from the previous frame to the current frame (i.e., negative or positive change in degree)<br>- stores wing angle velocities in new column<br>4) Returns updated coordinates.xlsx	os, pandas, numpy
6	near_ID.py	1) Prompts directory and loads coordinates.xlsx<br>2) Calculates and adds each of these variables:<br>- the distance (always based on thorax) between each male and all other males and females, identifying the male and the female nearest to it<br>- the distance to the nearest male and nearest female<br>- the change in distance to the nearest male and female compared to the previous frame<br>- approaching: the distance moved toward (or away from) the nearest male's or female's position in the previous frame (approaching or moving away)<br>3) Returns updated coordinates.xlsx	os, pandas, numpy

Table S5 (part 2/2)

7	*gaze_directions.py*	*1) Prompts directory and loads coordinates.xlsx*<br>*2) Calculates for each male:*<br>- *Gaze directions: the vector going from the thorax through the head, or if head not available, from the midpoint of the two ings through the thorax, or if thorax not available from the midpoint of the wing through the head*<br>- *Additional gaze vectors: rotates the gaze vector by 5 and 10 degrees on both sides, adds those*<br>- *Calculates the difference between the gaze angle of the previous and current frame*<br>- *Calculates the difference in gaze angle between the focal male and the nearest male and female*<br>*3) Returns updated coordinates.xlsx*	*pandas, numpy*
8	looking.py	1) Prompts directory and loads coordinates.xlsx<br>2) Determines fore each male:<br>- Whether he is looking at any other male or female, defined as any of the gaze vectors (main or additional) intersecting with any of the lines connecting the other individuals body parts<br>- Whether he is looking at the nearest male or female<br>- Gaze vector count for any male and female: how many of the total five gaze vectors go through any of the other individuals body lines<br>- Gaze vector count for nearest male and female<br>3) Returns updated coordinates.xlsx	pandas, numpy, os, math
9	velocities.py	1) Prompts directory and loads coordinates.xlsx<br>2) Calculates:<br>- Velocity (i.e. distance moved) between the previous and current frame for every individual<br>- Male and female directedness for every male: divides its "approaching" value calculated in the near_ID.py script by its velocity, to determin how much of the move in that frame was directed towards a male/female<br>3) Returns updated coordinates.xlsx	pandas, numpy, os
combined	master1.py	1) Runs script 1-9 from this Table on each of the 40 units automatically	os, subprocess

Table S6: Overview of python pipeline for processing data after training the models to predict behaviors.

	Python file	***Content***	***Libraries***
1	pred_file.py	1) Prompts directory and loads coordinates.xlsx<br>2) Creates a new excel file with the features used for behavioral prediction later as column names (full list available in Table S5)<br>3) Creates concatenated identifier for each combination of the unit, individual and frame to allow long format<br>4) Populates the empty frame with the corresponding data from coordinates.xlsx<br>5) Additionally calculates new features (using the file feature_calc.py) that were also used to train the models (as listed in Table S5)<br>6) Saves new file as filled_dataset_engineered.xlsx	pandas, numpy, os, re, subprocess, pexpect
2	predict.py	1) Loads trained models for mating, courtship and aggression detection<br>2) Loads from prompted file path filled_dataset_engineered.xlsx and makes predictions based on it, ignoring the features in each model that are not needed<br>3) Saves predictions and prediction scores as predicted_data.xlsx	pandas, numpy, os, joblib, sklearn
3	pred_back.py	1) Prompts for folder path<br>2) Puts the predictions back into the coordinates.xlsx file according to their identifiers	os, pandas
4	post_pred.py	1) Prompts directory and loads coordinates.xlsx<br>2) Corrects the mating predictions: as matings consist of long sequences of mating frames, wrong predictions in the middle of them might interrupt them and make it more difficult to count the matings. Corrections are made following these rules:<br>- First, single frame gaps are filled<br>- Second, small gaps (2 to 5 frames) are filled, depending on the density of 1's in the ten frames before and after each frame of the gap (filled if all frames of the gap have densities of 1's > 0.6)<br>- Then, larger gaps (6 to 10 frames) are filled, again depending on the density of 1's in the ten frames before and after each frame of the gap (filled if all frames of the gap have densities of 1's > 0.5)<br>- Each sequence after gap filling has to be > 80 frames or will be eliminated (as matings usually take between 3 and 7 minutes, 80 frames ≈160 seconds)<br>3) After mating corrections, predicted instances of courtship and aggression that occur during a male's mating are eliminated<br>4) The corrected predictions are saved to coordinates.xlsx	pandas, numpy, os
5	fem_mating.py	1) Prompts directory and loads coordinates.xlsx<br>2) Identifies matings in male's corrected mating predictions (as sequences of 1)<br>3) Constructs new columns for each female for their corrected mating predictions<br>4) For each identified mating sequence, determines the female that is closest to the male during that sequence for most of the frames, and adds the mating sequence to that females corrected mating prediction<br>5) Saves corrected predictions to coordinates.xlsx	os, pandas
combined	master2.py	1) Runs scripts 1-5 from this Table on each of the 40 units automatically	os, subprocess
After master2.py	summary_1.py and summary_2.py	1) After running master2.py, they summarize the data for each individual of each of the 40 units, leading to the data for analysis<br>2) For mating success: adds mates, length of mating sequences, so sequences shorter than 100 frames can be checked manually	os, pandas, tqdm, concurrent.futures, numpy

Table S7: Overview of hyperparameters and feature numbers, used in grid search and final random forest classifier models

Model	*Hyperparameter / Feature number*	*Explanation*	*tested*	*used in final model*
mating	**n_estimators**	the number of trees created by the random forest classifier	300, 350, 400, 450, 500	450
	**criterion**	how to decide on where to split the decision tree: based on gini or entropy, trying to minimize gini or entropy	gini, entropy	entropy
	**max_depth**	Maximum depth of each tree, maximum number of nodes in a tree. More nodes allow for more complex relationships	10, 20, no maximum	No maximum
	**min_samples_split**	minimum number samples required for a node to be able to split off on the tree	2,3,4,5	3
	**max_features**	maximum features used in a split (sqrt, 0.1 or 0.2 of feature number)	sqrt, 0.1, 0.2	sqrt
	**bootstrap**	whether to only use original data (bootstrapping = False) or bootstrap different subset of data for every tree	True, False	False
	**feature number**	number of features ending up in the final model, while eliminating those least useful to the model, narrowed down in each round	round 1: 85, 95, 105,115,125, 135<br>round 2: 45, 55, 65, 75, 85, 95, 105, 115, 125,135<br>round 3: 15, 25, 35, 45<br>round 4: 25, 30, 35, 40<br>round 5: 26, 28, 30, 32, 34<br>round 6: 28, 29, 30	30 (list of features in Table S6)
courtship	**n_estimators**	see above	300, 350, 400, 450, 500	400
	**criterion**	see above	gini, entropy	entropy
	**max_depth**	see above	10, 20, no maximum	No maximum
	**min_samples_split**	see above	2,3,4,5	2
	**max_features**	see above	sqrt, 0.1, 0.2	sqrt
	**bootstrap**	see above	True, False	False
	**feature number**	see above	round 1: 65, 100, 135<br>round 2: 15, 35, 55, 75<br>round 3: 45, 50, 55, 60, 65<br>round 4: 40, 45, 50<br>round 5: 40, 42, 45, 48, 50<br>round 6: 43, 44, 45, 46, 47, 48	47 (list of features in Table S6)
aggression	**n_estimators**	see above	300, 350, 400, 450, 500	300
	**criterion**	see above	gini, entropy	entropy
	**max_depth**	see above	10, 20, no maximum	no maximum
	**min_samples_split**	see above	2,3,4,5	2
	**max_features**	see above	sqrt, 0.1, 0.2	sqrt
	**bootstrap**	see above	True, False	False
	**feature number**	see above	round 1: 5, 15, 25, 35, 45, 55, 65, 75, 85, 95, 105, 115, 125, 135<br>round 2: 115, 125, 135<br>round 3: 110, 115, 120, 125<br>round 4: 110, 112, 115, 118, 120<br>round 5: 110,111,112,113,114, 115<br>round 6: 110, 111, 112	111 (list of features in Table S6)

Table S8 (part 1/4): Initial list of features used for training the random forest classifiers with explanations. Refer to Table S8 for a list of the features used in each of the final models and their relative importance

# Feature	*Feature*	*Explanation*
1	wing angle	angle between wings of focal
2	wing angle last	wing angle in previous frame
3	wing angle next	wing angle in next frame
4	nearest male distance	distance to nearest male
5	nearest male distance last	distance to nearest male previous frame
6	nearest male distance next	distance to nearest male next frame
7	nearest female distance	distance to nearest female
8	nearest female distance last	distance to nearest female previous frame
9	nearest female distance next	distance to nearest female next frame
10	male distance change	change in distance to nearest male
11	male distance change last	change in distance to nearest male in previous frame
12	male distance change next	change in distance to nearest male in next frame
13	female distance change	change in distance to nearest female
14	female distance change last	change in distance to nearest female in previous frame
15	female distance change next	change in distance to nearest female in next frame
16	approaching male	approaching (distance change) nearest male's previous position
17	approaching male last	approaching male in previous frame
18	approaching male next	approaching male in next frame
19	approaching female	approaching (distance change) nearest fefmale's previous position
20	approaching female last	approaching female in previous frame
21	approaching female next	approaching female in next frame
22	turning angle	angle by which gaze direction of focal turned
23	turning angle last	turning angle in previous frame
24	turning angle next	turning angle in next frame
25	nearest male gaze difference	angle between focal and nearest male's gaze directions
26	nearest male gaze difference last	angle between focal and nearest male's gaze directions in previous frame
27	nearest male gaze difference next	angle between focal and nearest male's gaze directions in next frame
28	nearest female gaze difference	angle between focal and nearest female's gaze directions
29	nearest female gaze difference last	angle between focal and nearest female's gaze directions in previous frame
30	nearest female gaze difference next	angle between focal and nearest female's gaze directions in next frame
31	looking at male	whether the focal is looking at any male
32	looking at male last	whether the focal is looking at any male in previous frame
33	looking at male next	whether the focal is looking at any male in next frame
34	looking at female	whether the focal is looking at any female
35	looking at female last	whether the focal is looking at any female in previous frame
36	looking at female next	whether the focal is looking at any female in next frame

Table S8 (part 2/4)

# Feature	*Feature*	*Explanation*
37	looking at nearest male	whether the focal is looking at the nearest male
38	looking at nearest male last	whether the focal is looking at the nearest male in previous frame
39	looking at nearest male next	whether the focal is looking at the nearest male in next frame
40	looking at nearest female	whether the focal is looking at the nearest female
41	looking at nearest female last	whether the focal is looking at the nearest female in previous frame
42	looking at nearest female next	whether the focal is looking at the nearest female in next frame
43	velocity	focal's distance moved since last frame
44	velocity last	velocity in previous frame
45	velocity next	velocity in next frame
46	male directedness	proportion of velocity explained by approaching male
47	male directedness last	male directedness in previous frame
48	male directedness next	male directedness in next frame
49	female directedness	proportion of velocity explained by approaching female
50	female directedness last	female directedness in previous frame
51	female directedness next	female directedness in next frame
52	wing angle velocity	change in wing angle from previous frame
53	wing angle velocity last	wing angle velocity in previous frame
54	wing angle velocity next	wing angle velocity in last frame
55	gaze vector count nearest male	number of gaze vectors intersecting the nearest male
56	gaze vector count nearest male last	number of gaze vectors intersecting the nearest male in previous frame
57	gaze vector count nearest male next	number of gaze vectors intersecting the nearest male in next frame
58	gaze vector count nearest female	number of gaze vectors intersecting the nearest female
59	gaze vector count nearest female last	number of gaze vectors intersecting the nearest female in previous frame
60	gaze vector count nearest female next	number of gaze vectors intersecting the nearest female in next frame
61	gaze vector count male	number of gaze vectors intersecting any male
62	gaze vector count male last	number of gaze vectors intersecting any male in previous frame
63	gaze vector count male next	number of gaze vectors intersecting any male in next frame
64	gaze vector count female	number of gaze vectors intersecting any female
65	gaze vector count female last	number of gaze vectors intersecting any female in previous frame
66	gaze vector count female next	number of gaze vectors intersecting any female in next frame
67	distance nearest male and female	distance between the nearest male and nearest female
68	distance nearest male and female last	distance between the nearest male and nearest female in previous frame
69	distance nearest male and female next	distance between the nearest male and nearest female in next frame

Table S8 (part 3/4)

# Feature	*Feature*	*Explanation*
70	velocity ^{2}	velocity squared
71	velocity ^{3}	velocity to the power of 3
72	velocity ^{4}	velocity to the power of 4
73	velocity^{5}	velocity to the power of 5
74	log velocity	$\log_{10}$(velocity)
75	vector difference	difference in gaze vector count between nearest male and nearest female
76	head / wing angle	angle between female's wings and focal male's head, with the head as the midpoint
77	head wing angle last	head / wing angle in previous frame
78	head wing angle next	head / wing angle in next frame
79	interaction 1	feature 67 * feature 68
80	interaction 2	feature 67 * feature 69
81	interaction 3	feature 4 * feature 6
82	interaction 4	feature 4 * feature 5
83	interaction 5	feature 7 * feature 68
84	interaction 6	feature 7 * feature 69
85	interaction 7	feature 7 * feature 67
86	interaction 8	feature 13 * feature 49
87	interaction 9	feature 16 * feature 49
88	interaction 10	feature 16 * feature 22
89	interaction 11	feature 16 * feature 45
90	interaction 12	feature 16 * feature 74
91	interaction 13	feature 16 * feature 43
92	interaction 14	feature 19 * feature 49
93	interaction 15	feature 22 * feature 43
94	interaction 16	feature 22 * feature 24
95	interaction 17	feature 25 * feature 27
96	interaction 18	feature 25 * feature 26
97	interaction 19	feature 28 * feature 60
98	interaction 20	feature 28 * feature 59
99	interaction 21	feature 28 * feature 58
100	interaction 22	feature 31 * feature 63
101	interaction 23	feature 31 * feature 32
102	interaction 24	feature 31 * feature 33
103	interaction 25	feature 34 * feature 36
104	interaction 26	feature 34 * feature 35
105	interaction 27	feature 34 * feature 75
106	interaction 28	feature 34 * feature 65
107	interaction 29	feature 34 * feature 66
108	interaction 30	feature 34 * feature 41
109	interaction 31	feature 34 * feature 59

Table S8 (part 4/4)

# Feature	*Feature*	*Explanation*
110	interaction 32	feature 37 * feature 55
111	interaction 33	feature 37 * feature 41
112	interaction 34	feature 37 * feature 57
113	interaction 35	feature 37 * feature 63
114	interaction 36	feature 37 * feature 42
115	interaction 37	feature 40 * feature 59
116	interaction 38	feature 40 * feature 41
117	interaction 39	feature 40 * feature 42
118	interaction 40	feature 40 * feature 42
119	interaction 41	feature 40 * feature 65
120	interaction 42	feature 40 * feature 60
121	interaction 43	feature 40 * feature 66
122	interaction 44	feature 43 * feature 69
123	interaction 45	feature 43 * feature 67
124	interaction 46	feature 43 * feature 44
125	interaction 47	feature 43 * feature 45
126	interaction 48	feature 55 * feature 63
127	interaction 49	feature 55 * feature 57
128	interaction 50	feature 58 * feature 67
129	interaction 51	feature 58 * feature 69
130	interaction 52	feature 58 * feature 68
131	interaction 53	feature 58 * feature 59
132	interaction 54	feature 58 * feature 60
133	interaction 55	feature 58 * feature 65
134	interaction 56	feature 61 * feature 62
135	interaction 57	feature 64 * feature 70 * feature 4
136	interaction 58	feature 64 * feature 65
137	interaction 59	feature 64 * feature 66
138	interaction 60	feature 67 * feature 74

Table S9 part (1/2): List of selected features for each model, sorted by feature importance (imp.)

Mating		***courtship***		***aggression***	
imp.	**feature**	**imp.**	**feature**	**imp.**	**feature**
0.1468	nearest female distance	0.0844	nearest female distance	0.0685	interaction 3
0.1289	nearest female distance last	0.0739	nearest female distance last	0.0618	nearest male distance
0.1176	nearest female distance next	0.0485	nearest female distance next	0.0458	interaction 4
0.1054	head / wing angle last	0.0443	interaction 57	0.0413	nearest male distance next
0.1045	head / wing angle next	0.0316	head / wing angle last	0.0369	interaction 47
0.0628	head / wing angle	0.0315	interaction 47	0.0309	interaction 7
0.0249	interaction 6	0.0298	head / wing angle	0.0303	interaction 46
0.0243	interaction 7	0.0293	interaction 21	0.0254	nearest male distance last
0.0205	distance nearest male and female next	0.0287	vector difference	0.0212	interaction 11
0.0201	interaction 46	0.0276	interaction 46	0.0208	$velocity^2$
0.0186	interaction 47	0.0264	velocity next	0.0198	approaching male
0.0181	interaction 7	0.0256	interaction 27	0.0197	log velocity
0.0165	distance nearest male and female last	0.0239	interaction 20	0.0183	$velocity^4$
0.0145	interaction 21	0.0234	gaze vector count nearest female	0.0179	distance nearest male and female
0.0145	wing angle	0.0228	interaction 52	0.0177	interaction 13
0.0139	velocity last	0.0221	head / wing angle next	0.0169	interaction 12
0.0134	wing angle next	0.0217	looking at nearest female	0.0167	interaction 32
0.0126	wing angle last	0.0190	interaction 7	0.0159	velocity next
0.0125	distance nearest male and female	0.0184	gaze vector count female last	0.0157	$velocity^5$
0.0115	interaction 58	0.0183	velocity last	0.0155	$velocity^3$
0.0113	velocity next	0.0177	interaction 5	0.0145	interation 45
0.0099	nearest female gaze difference next	0.0177	interaction 50	0.0139	looking at nearest male
0.0095	nearest female gaze difference last	0.0164	gaze vector count nearest female last	0.0137	gaze vector count nearest male
0.0094	approaching female last	0.0149	interaction 58	0.0137	interaction 60
0.0091	nearest female gaze difference	0.0141	distance nearest male and female	0.0131	vector difference
0.0089	female distance change last	0.0135	distance nearest male and female last	0.0125	gaze vector count male
0.0076	interaction 59	0.0133	gaze vector count nearest female next	0.0124	velocity
0.0073	female distance change next	0.0131	interaction 45	0.0117	interaction 10
0.0071	interaction 31	0.0127	interaction 19	0.011	velocity last
0.007	interaction 28	0.0124	$velocity^2$	0.0102	interaction 6
0.0066	gaze vector count nearest female last	0.0121	interaction 6	0.0099	distance nearest male and female next
0.0046	gaze vector count female next	0.0120	interaction 60	0.0096	approaching male last
		0.0114	$velocity^3$	0.0095	interaction 44
		0.0108	$velocity^4$	0.0076	interaction 15
		0.0106	nearest female gaze difference last	0.0071	interaction 5
		0.0106	wing angle last	0.007	male directedness
		0.0105	wing angle next	0.0068	approaching male next
		0.0104	$velocity^5$	0.0067	distance nearest male and female last
		0.0102	interaction 44	0.0065	nearest female gaze difference
		0.0101	velocity	0.0064	interaction 9
		0.0101	nearest female gaze difference	0.006	nearest female distance
		0.0100	turning angle next	0.006	nearest female gaze difference last
		0.0099	wing angle	0.0056	nearest female gaze difference next
		0.0098	gaze_vector_count_female_next	0.0055	male directedness last
		0.0094	approaching female last	0.0055	wing angle next
		0.0092	interaction 4	0.0054	interaction 48
		0.0091	interaction 16	0.0054	looking at male
		0.0091	nearest male distance last	0.0052	turning angle last
		0.0090	log velocity	0.0051	gaze vector count male next
		0.008465	nearest female gaze difference next	0.005	wing angle

Table S9 part (2/2)

aggression	
imp.	**feature**
0.005	interaction 2
0.0048	male directedness next
0.0048	wing angle last
0.0048	gaze vector count male last
0.0047	gaze vector count nearest male next
0.0046	interaction 16
0.0045	turning angle next
0.0045	approaching female next
0.0044	nearest female distance next
0.0043	male distance change next
0.0043	turning angle
0.0042	interaction 50
0.0042	female distance change next
0.0042	nearest male gaze difference next
0.0042	nearest female distance last
0.004	interaction 1
0.0039	approaching female last
0.0038	gaze vector count nearest male last
0.0038	male distance change last
0.0038	male distance change
0.0037	interaction 56
0.0037	approaching female
0.0036	female distance change
0.0036	interaction 52
0.0035	wing angle velocity next
0.0035	interaction 17
0.0034	looking at nearest male next
0.0033	female distance change last
0.0033	female directedness
0.0033	nearest male gaze difference
0.0033	interaction 35
0.0033	female directedness next
0.0032	wing angle velocity last
0.0032	interaction 18
0.0032	female directedness last
0.0031	looking at nearest male last
0.0031	interaction 8
0.0031	interaction 14
0.003	wing angle velocity
0.0029	nearest male gaze difference last
0.0026	interaction 51
0.0021	interaction 20
0.002	interaction 21
0.0019	interaction 57
0.0017	interaction 19
0.0017	interaction 22
0.0017	interaction 58
0.0016	gaze vector count female next
0.0016	interaction 59
0.0015	interaction 28
0.0014	looking at male last
0.0014	interaction 29
0.0013	interaction 27
0.0012	gaze vector count female last
0.0012	gaze vector count female
0.0012	interaction 53
0.0012	interaction 55
0.0011	interaction 41
0.0011	interaction 43
0.001	interaction 54
0.0009	interaction 37

Table S10: Performance of models on validation set after training.

	precision (negative class)	*recall (negative class)*	*precision (positive class)*	*recall (positive class)*	*model accuracy*
mating	0.9764	0.9837	0.9840	0.9769	0.9802
courtship	0.9463	0.9090	0.9180	0.9517	0.9311
aggression	0.9490	0.9254	0.9333	0.9545	0.9406

Table S11: Ethogram of male aggression behaviors, which was the basis for labeling the training data for categorization of aggression

**Behavior**	**Definition**
Lunging	Rearing up and collapsing onto opponent
Chasing	Running after opponent
Head butt	Thrusting head into opponents body
Tussling	Tumbling over opponent
Fencing	Pushing opponents leg
Boxing	Striking/shoving opponent with forelegs while both are reared on hindlegs

Table S12: Summary table for different models on total RDA occupancy (i.e., proportion of frames in RDAs; overall or non-mating frames; with or without buffer around small RDA), including effect size with 95% confidence interval.

**Model**	**Predictor**	**Est.**	**SE**	**z**	**P**	**Odds Ratio [95% CI]**
Overall & without buffer	(Intercept)	-1.09	0.07	-14.67	**< 0.001**	**0.34 [0.29, 0.39]**
	Sex (M)	0.25	0.08	3.00	**0.003**	**1.29 [1.09, 1.52]**
Overall & with buffer	(Intercept)	-0.74	0.07	-10.07	**< 0.001**	**0.48 [0.41, 0.55]**
	Sex (M)	0.23	0.08	2.82	**0.005**	**1.26 [1.07, 1.48]**
Non-mating & without buffer	(Intercept)	-1.15	0.07	-15.52	**< 0.001**	**0.32 [0.27, 0.36]**
	Sex (M)	0.34	0.08	4.07	**< 0.001**	**1.41 [1.20, 1.66]**
Non-mating & with buffer	(Intercept)	-0.78	0.07	-10.55	**< 0.001**	**0.46 [0.40, 0.53]**
	Sex (M)	0.30	0.08	3.66	**< 0.001**	**1.35 [1.15, 1.59]**

'Buffer' refers to the buffer area surrounding the small RDABold values indicate p < 0.05.

Table S13a and b: ANOVA (a) and summary table (b) of occupancy (proportion of frames in RDAs) depending on sex, RDA size (large, small and small with buffer) and quality, and the quality of the other RDA.

(a)

Predictor	χ^2	df	P
Sex	16.60	1	**< 0.001**
RDA quality	9.72	1	**0.002**
RDA size	384.68	2	**< 0.001**
RDA quality other	5.47	1	**0.019**
RDA quality × RDA size	1.16	2	0.559
RDA quality × RDA quality other	2.94	1	0.086
RDA size × RDA quality other	0.24	2	0.885
RDA quality × RDA size × RDA quality other	18.74	2	**< 0.001**

Bold values indicate P < 0.05.

(b)

Predictor	Est.	SE	z	P	Odds Ratio [95% CI]
Intercept	-1.30	0.14	-9.22	**< 0.001**	**0.27 [0.21, 0.36]**
Sex (M)	0.27	0.07	4.07	**< 0.001**	**1.30 [1.15, 1.48]**
RDA quality (Low)	-0.50	0.19	-2.56	**0.010**	**0.61 [0.41, 0.89]**
RDA size (buffer)	-1.16	0.15	-7.99	**< 0.001**	**0.31 [0.23, 0.42]**
RDA size (small)	-1.89	0.16	-11.99	**< 0.001**	**0.15 [0.11, 0.21]**
RDA quality other (Low)	0.06	0.19	0.30	0.768	1.06 [0.73, 1.54]
RDA quality (Low) × RDA size (buffer)	0.63	0.24	2.57	**0.010**	**1.88 [1.16, 3.03]**
RDA quality (Low) × RDA size (small)	0.76	0.26	2.95	**0.003**	**2.13 [1.29, 3.52]**
RDA quality (Low) × RDA quality other (Low)	0.25	0.27	0.93	0.355	1.29 [0.75, 2.20]
RDA size (buffer) × RDA quality other (Low)	0.63	0.24	2.60	**0.009**	**1.87 [1.17, 3.01]**
RDA size (small) × RDA quality other (Low)	0.59	0.25	2.32	**0.021**	**1.80 [1.09, 2.95]**
RDA quality (Low) × RDA size (buffer) × RDA quality other (Low)	-1.07	0.28	-3.79	**< 0.001**	**0.34 [0.20, 0.59]**
RDA quality (Low) × RDA size (small) × RDA quality other (Low)	-1.07	0.30	-3.56	**< 0.001**	**0.34 [0.19, 0.62]**

'Buffer' refers to the buffer area surrounding the small RDA. Bold values indicate p < 0.05.

Table S14. Mean Jacobs' index values (± 95% bootstraped confidence intervals) describing preference (> 0) or avoidance (< 0) of RDAs depending on size and quality in a) males and b) females. Each table shows data across all individuals of that sex and across only those that were detected in an RDA for at least one frame (i.e., Jacobs' index > -1). One-sample tests evaluate whether mean Jacobs' index values differed from zero; significant P-values are shown in bold.

a)

Facet	RDA size	RDA quality	Other RDA quality	n	Mean [95% CI]	t	P
All males	Large	High	High	80	0.383 [0.239, 0.514]	5.34	**< 0.001**
All males	Large	High	Low	80	0.437 [0.310, 0.553]	7.04	**< 0.001**
All males	Large	Low	High	79	0.207 [0.060, 0.341]	2.87	**0.005**
All males	Large	Low	Low	80	0.289 [0.136, 0.425]	4.02	**< 0.001**
All males	Small	High	High	80	-0.398 [-0.561, -0.225]	-4.83	**< 0.001**
All males	Small	High	Low	79	-0.011 [-0.178, 0.160]	-0.12	0.902
All males	Small	Low	High	80	-0.269 [-0.419, -0.119]	-3.38	**0.001**
All males	Small	Low	Low	80	-0.362 [-0.525, -0.197]	-4.36	**< 0.001**
Only males with recorded RDA use	Large	High	High	71	0.558 [0.454, 0.649]	10.85	**< 0.001**
Only males with recorded RDA use	Large	High	Low	73	0.575 [0.492, 0.650]	14.30	**< 0.001**
Only males with recorded RDA use	Large	Low	High	68	0.402 [0.295, 0.507]	7.44	**< 0.001**
Only males with recorded RDA use	Large	Low	Low	71	0.452 [0.339, 0.555]	8.04	**< 0.001**
Only males with recorded RDA use	Small	High	High	39	0.235 [0.052, 0.417]	2.56	**0.015**
Only males with recorded RDA use	Small	High	Low	57	0.371 [0.222, 0.505]	5.20	**< 0.001**
Only males with recorded RDA use	Small	Low	High	49	0.194 [0.042, 0.339]	2.61	**0.012**
Only males with recorded RDA use	Small	Low	Low	39	0.308 [0.142, 0.455]	3.84	**< 0.001**

b)

Facet	RDA size	RDA quality	Other RDA quality	n	Mean [95% CI]	t	P
All females	Large	High	High	80	0.191 [0.037, 0.341]	2.41	**0.018**
All females	Large	High	Low	80	0.206 [0.042, 0.350]	2.65	**0.010**
All females	Large	Low	High	80	-0.125 [-0.270, 0.021]	-1.61	0.111
All females	Large	Low	Low	80	0.069 [-0.078, 0.216]	0.92	0.360
All females	Small	High	High	80	-0.464 [-0.631, -0.304]	-5.33	**< 0.001**
All females	Small	High	Low	80	-0.165 [-0.348, 0.014]	-1.68	0.098
All females	Small	Low	High	80	-0.446 [-0.588, -0.295]	-5.88	**< 0.001**
All females	Small	Low	Low	80	-0.491 [-0.631, -0.344]	-6.54	**< 0.001**
Only females with recorded RDA use	Large	High	High	70	0.361 [0.226, 0.493]	5.21	**< 0.001**
Only females with recorded RDA use	Large	High	Low	68	0.418 [0.298, 0.537]	6.73	**< 0.001**
Only females with recorded RDA use	Large	Low	High	60	0.167 [0.040, 0.302]	2.39	**0.020**
Only females with recorded RDA use	Large	Low	Low	71	0.205 [0.067, 0.339]	2.94	**0.004**
Only females with recorded RDA use	Small	High	High	32	0.340 [0.095, 0.557]	2.93	**0.006**
Only females with recorded RDA use	Small	High	Low	42	0.591 [0.436, 0.734]	7.55	**< 0.001**
Only females with recorded RDA use	Small	Low	High	40	0.108 [-0.059, 0.274]	1.25	0.220
Only females with recorded RDA use	Small	Low	Low	38	0.072 [-0.111, 0.255]	0.76	0.453

Table S15: Summary table of average number of males in RDAs depending on size and quality (n = 80; i.e., one observation per RDA per arena). r denotes a correlation-like effect size derived from the t statistic.

Predictor	Est.	SE	t	P	r
Intercept	2.20	0.13	17.34	**< 0.001**	**0.89**
RDA size (Small)	-1.09	0.15	-7.45	**< 0.001**	**-0.65**
Quality (Low)	-0.44	0.15	-3.00	**0.004**	**-0.32**

Bold values indicate p < 0.05.

Table S16: Summary table of proportion exclusive occupancy of RDAs (i.e., proportion of frames in which a male has no competitor in an RDA) depending on size and quality (*n* = 80; i.e., one observation per RDA per arena)

Predictor	Est.	SE	z	P	Odds Ratio [95% CI]
Intercept	-1.02	0.19	-5.42	**< 0.001**	**0.36 [0.25, 0.52]**
RDA size (Small)	1.35	0.21	6.35	**< 0.001**	**3.87 [2.55, 5.87]**
Quality (Low)	0.58	0.21	2.77	**0.006**	**1.79 [1.18, 2.70]**

Bold values indicate p < 0.05.

Table S17: Summary table of average number of accesses in RDAs (i.e., times a male enters an RDA) depending on size and quality (*n* = 638; i.e., one observation per RDA per arena)

Predictor	Est.	SE	z	P	Rate Ratio [95% CI]
Intercept	1.39	0.10	13.69	**< 0.001**	**4.02 [3.29, 4.90]**
RDA size (Large)	1.12	0.10	11.71	**< 0.001**	**3.06 [2.53, 3.68]**
Quality (High)	0.14	0.11	1.28	0.201	1.15 [0.93, 1.41]

Bold values indicate p < 0.05.

Table S18: Summary table on male occupancy of the large RDA across the full 30-minute trial (*n* = 319). Predictor variable mean-standardized per arena.

Predictor	Est.	SE	z	P	Odds Ratio [95% CI]
Intercept	-1.32	0.09	-15.35	**< 0.001**	**0.27 [0.23, 0.32]**
Occupancy-S	-0.63	0.07	-8.55	**< 0.001**	**0.54 [0.46, 0.62]**
Aggression	0.52	0.07	6.94	**< 0.001**	**1.68 [1.45, 1.94]**
Mobility	0.18	0.07	2.46	**0.014**	**1.20 [1.04, 1.39]**

Bold values indicate p < 0.05.

Table S19: Summary table of LMM on male occupancy of the small RDA across the full 30-minute trial (*n* = 319). Response and predictor variable mean-standardized per arena.

Predictor	Est.	SE	z	P	Odds Ratio [95% CI]
Intercept	-2.47	0.17	-14.75	**< 0.001**	**0.08 [0.06, 0.12]**
Occupancy-L	-0.67	0.09	-7.30	**< 0.001**	**0.51 [0.43, 0.61]**
Aggression	0.28	0.10	2.95	**0.003**	**1.33 [1.10, 1.60]**
Mobility	0.18	0.09	1.98	**0.048**	**1.19 [1.00, 1.42]**

Bold values indicate p < 0.05.

Table S20a and b: ANOVA (a) and summary table (b) on the RDA occupancy (proportion of frames in RDAs) in males across time periods

(a)

**Predictor**	**χ^2**	**df**	**P**
Occupancy of other RDA	846.29	1	**< 0.001**
RDA size	157.40	1	**< 0.001**
Aggression	364.44	1	**< 0.001**
Number of competitors in RDA	0.74	1	0.389
Number of females in RDA	75.89	1	**< 0.001**
Mobility	41.05	1	**< 0.001**
RDA quality	46.30	1	**< 0.001**
RDA size × Aggression	16.69	1	**< 0.001**
Aggression × Number of competitors in RDA	275.69	1	**< 0.001**
Aggression × Number of females in RDA	17.03	1	**< 0.001**

Bold values indicate p < 0.05.

(b)

**Predictor**	**Est.**	**SE**	**z**	**P**	**Odds Ratio [95% CI]**
Intercept	-0.99	0.12	-7.97	**< 0.001**	**0.37 [0.29, 0.48]**
Occupancy of other RDA	-9.46	0.33	-29.09	**< 0.001**	**0.00 [0.00, 0.00]**
RDA size (Small)	-0.49	0.04	-12.36	**< 0.001**	**0.61 [0.57, 0.66]**
Aggression	0.45	0.02	18.29	**< 0.001**	**1.57 [1.50, 1.65]**
Number of competitors in RDA	-0.06	0.02	-2.77	**0.006**	**0.94 [0.91, 0.98]**
Number of females in RDA	0.17	0.02	8.97	**< 0.001**	**1.18 [1.14, 1.22]**
Mobility	0.14	0.02	6.41	**< 0.001**	**1.15 [1.10, 1.19]**
Quality (Low)	-0.36	0.05	-6.80	**< 0.001**	**0.70 [0.63, 0.77]**
RDA size (Small) × Aggression	-0.15	0.04	-4.08	**< 0.001**	**0.86 [0.80, 0.92]**
Aggression × Number of competitors in RDA	0.32	0.02	16.60	**< 0.001**	**1.37 [1.32, 1.43]**
Aggression × Number of females in RDA	-0.08	0.02	-4.13	**< 0.001**	**0.93 [0.89, 0.96]**

Bold values indicate p < 0.05.

Table S21a and b: ANOVA (a) and summary table (b) on the RDA occupancy (proportion of frames in RDAs) in females across time periods

(a)

**Predictor**	**χ^2**	**df**	**P**
Occupancy other RDA	396.06	1	**< 0.001**
RDA size	26.37	1	**< 0.001**
Mating status	12.78	1	**< 0.001**
Number of other females in RDA	34.20	1	**< 0.001**
Number of males in RDA	45.41	1	**< 0.001**
Mobility	120.99	1	**< 0.001**
RDA quality	50.56	1	**< 0.001**
RDA size × Mating status	49.99	1	**< 0.001**

Bold values indicate $p < 0.05$.

(b)

**Predictor**	**Est.**	**SE**	**z**	**P**	**Odds Ratio [95% CI]**
Intercept	-1.10	0.13	-8.64	**< 0.001**	**0.33 [0.26, 0.43]**
Occupancy other RDA	-8.38	0.42	-19.90	**< 0.001**	**0.00 [0.00, 0.00]**
RDA size (Small)	-0.63	0.07	-8.67	**< 0.001**	**0.53 [0.46, 0.61]**
Mating status (virgin)	-0.52	0.09	-6.04	**< 0.001**	**0.60 [0.50, 0.71]**
Number of other females in RDA	0.14	0.02	5.85	**< 0.001**	**1.15 [1.10, 1.20]**
Number of males in RDA	0.17	0.02	6.74	**< 0.001**	**1.18 [1.13, 1.24]**
Mobility	0.22	0.02	11.00	**< 0.001**	**1.25 [1.20, 1.30]**
Quality (Low)	-0.46	0.07	-7.11	**< 0.001**	**0.63 [0.55, 0.71]**
RDA size (Small) × Mating status (virgin)	0.63	0.09	7.07	**< 0.001**	**1.89 [1.58, 2.25]**

Bold values indicate $p < 0.05$.
